# Multi‑strategy Equilibrium Optimizer: An improved meta-heuristic tested on numerical optimization and engineering problems

**DOI:** 10.1371/journal.pone.0276210

**Published:** 2022-10-20

**Authors:** Yu Li, Xiao Liang, Jingsen Liu, Huan Zhou

**Affiliations:** 1 Institute of Management Science and Engineering, Henan University, Kaifeng, China; 2 School of Business, Henan University, Kaifeng, China; 3 Institute of Intelligent Network Systems, and Software School, Henan University, Kaifeng, China; Nottingham Trent University School of Science and Technology, UNITED KINGDOM

## Abstract

The Equilibrium Optimizer (EO) is a recently proposed intelligent optimization algorithm based on mass balance equation. It has a novel principle to deal with global optimization. However, when solving complex numerical optimization problems and engineering problems, the algorithm will get stuck into local optima and degrade accuracy. To address the issue, an improved Equilibrium Optimizer (IEO) based on multi-strategy optimization is proposed. First, Tent mapping is used to generate the initial location of the particle population, which evenly distributes the particle population and lays the foundation for diversified global search process. Moreover, nonlinear time parameter is used to update the position equation, which dynamically balances the exploration and exploitation phases of improved algorithm. Finally, Lens Opposition‑based Learning (LOBL) is introduced, which avoids local optimization by improving the population diversity of the algorithm. Simulation experiments are carried out on 23 classical functions, IEEE CEC2017 problems and IEEE CEC2019 problems, and the stability of the algorithm is further analyzed by Friedman statistical test and box plots. Experimental results show that the algorithm has good solution accuracy and robustness. Additionally, six engineering design problems are solved, and the results show that improved algorithm has high optimization efficiency achieves cost minimization.

## 1. Introduction

In recent years, optimization problems have become an important topic in the modern management field. They help provide the optimal solution for the application problems in various fields of society. On the premise of comprehensive consideration of all aspects of constraints, the practical application problems are abstracted as objective functions and solved. Because of the development of science and technology, many optimization problems become more and more complex. Meta-heuristic algorithms with high flexibility have subsequently attracted the attention of a large number of researchers [1]. The meta-heuristic algorithms put forward a set of new research ideas and solutions, some in the modeling and simulation of complex systems, others analyzing complex decision and solving optimization problems. Meta-heuristic algorithms are used to solve optimization problems by simulating biological or physical phenomena. The algorithms are divided into four categories: evolutionary algorithms, physics-based algorithms, human-based algorithms and swarm intelligence algorithms [2].

Evolutionary algorithms realize the overall progress of the population and achieve the optimal solution by simulating the evolution law of survival of the fittest in nature. The representative ones include the genetic algorithm (GA) that searches for the optimal solution by simulating the natural evolution process [3], and the differential evolution (DE) that simulates the crossover and mutation mechanism in heredity [4]. Similarly, population-based incremental learning (PBIL) is proposed based on two strategies: genetic search and competitive learning [5]. Biogeography-based optimization (BBO) is proposed based on the biogeography theory of species’ migration and drift between geographical regions in nature [6].

Physics-based algorithms are inspired by the laws of physics in nature. Representative algorithms mainly include simulated annealing (SA) [7], whose inspiration comes from the annealing process of solid materials. In addition, big-bang big-crunch (BBBC) is inspired by the Big Bang and contraction theory [8]. Gravitational search algorithm (GSA) is based on Newton’s law of universal gravity to guide the motion of each particle to search for the optimal solution [9]. Inspired by the theory of physics kinematics, central force optimization (CFO) realizes the update of the optimal solution by updating the acceleration [10].

Human-based algorithms are modeled after human behaviors, such as human teaching behaviors and social behaviors. The representative algorithms are listed below. Tabu search (TS) is a search process guided by memory. It is also a simulation of human intelligence process and a manifestation of artificial intelligence [11]. Teaching learning based optimization (TLBO) conducts search optimization by simulating the learning methods of human teaching and techniques in the learning process [12]. The inspiration of harmony search (HS) comes from the process of human musical performance [13], by repeatedly adjusting the solution variables in the memory bank, the algorithm makes the function value continuously converge with the increase of the number of iterations.

The swarm intelligence algorithm comes from the simulation of biological groups’ behavior process. The individuals in the biological group follow the cooperative mode of aggregation, division of labor, collision avoidance, and convergence, until the swarm intelligence emerges. Representative swarm intelligence algorithms include: particle swarm optimization (PSO), which simulates the foraging behavior of birds [14]; ant colony optimization (ACO), which simulates the foraging path of ants by secretion concentration [15]; artificial bee colony (ABC), which simulates the honey gathering behavior of bees [16], and whale optimization algorithm (WOA), which is inspired by the feeding behavior of whales in the ocean [17].

Equilibrium Optimizer(EO) is a physics-based meta-heuristic algorithm proposed in 2020 [18], The algorithm was inspired by mass balance equations in physics. The EO algorithm is inspired by the control volume mass balance, where the dynamic state and equilibrium state of the particles can be estimated. In EO, the important parameters are: equilibrium pool (${\vec{C}}_{eq,pool}$), exponential term ($\vec{F}$) and generation rate ($\vec{GCP}$). During the optimization process, the search agent randomly updates its concentration (position) for certain particles called equilibrium pool, eventually reaching an equilibrium state (the best result). The unique update mechanism of EO algorithm makes it equipped with fast convergence speed and precision. Some scholars have studied EO algorithm are listed here. Gupta et al. [19] introduced Gaussian variation and new exploratory search mechanism on the basis of EO algorithm to improve the diversity of solutions. Abdel-Basset et al. [20] introduced an equation and Gaussian mutation strategy when EO was performing position update to improve the exploration and exploitation capability of the algorithm. Jia et al. [21] combined EO with thermal exchange optimization (TEO) in order to improve the optimization accuracy of EO algorithm. Fan et al. [1] proposed an improved EO algorithm based on opposition-based learning and new update rules, which improve the exploration ability of the algorithm and avoid falling into local optimal value.

EO has a competitive advantage compared with other intelligent optimization algorithms. Still, it has problems such as slow convergence speed, low solution accuracy and tendency to fall into local optima when solving complex function and engineering problems. These defects are mainly caused by the following reasons: low quality of randomly generated initial particle population, unbalanced exploration and exploitation abilities in the iteration phase, and the decrease of population diversity caused by particle aggregation in the later iteration phase. Therefore, an improved Equilibrium Optimizer (IEO) based on multi-strategy optimization is proposed. It includes three improvements: first, the random initialization is replaced by a Tent chaotic map, so the particles are evenly distributed in the search space as far as possible, and the quality of the initial solution is improved. Second, a dynamic control parameter strategy is proposed to promote the balance between the exploration and exploitation phases of the algorithm through the dynamic changes of parameters. Finally, the Lens Opposition‑based Learning (LOBL) strategy is introduced in a late iteration, which prevents the algorithm from falling into local optimum by finding other valuable search areas through generating new candidate solutions. In the simulation experiment, three different complexity test sets are optimized, namely 23 reference functions, IEEE CEC2017 and IEEE CEC2019 test sets. When all the experimental results are compared with the six meta-heuristic algorithms, the results show that the improved algorithm IEO has significant advantages in convergence accuracy and effectiveness. Among statistical tests, the Friedman test and the Wilcoxon rank sum test prove that IEO has superior performance when optimizing each test set. In addition, this paper also selected a convergence curve and boxplot analysis to show the stability of IEO from different perspectives. Finally, the improved algorithm IEO is applied to six engineering design problems: the pressure vessel problem, the welded beam problem, the tension/compression spring problem, the three-bar truss problem, the speed reducer problem and optimal design of industrial refrigeration system. Experimental results show that IEO has good optimization efficiency in solving practical application problems. With knowledge of above discussion, the innovations of this paper are listed as follows.

- To improve the quality of the initial particle population, Tent chaotic mapping is introduced to increase the diversity of initial solutions through the map’s uniformity distribution.
- A new dynamic control parameter strategy is proposed to facilitate an effective transition from exploration to exploitation, to improve search efficiency and avoid premature convergence.
- To avoid falling into a local optimal solution due to population aggregation phenomenon at the later stage of iterations, a LOBL strategy is introduced to expand the search space and improve the convergence speed.
- Experiments are carried out on three function test sets of different difficulty, namely 23 benchmark functions, IEEE CEC2017 and IEEE CEC2019. IEO has the advantages of high solution accuracy, fast convergence speed and strong robustness when optimizing complex functions.
- The effectiveness and optimization efficiency of IEO are tested on six engineering problems of different complexity.
- This paper includes two different types of experiments, the first is about the analysis of numerical experimental results, and the second is about the application of engineering problems. Two different types of tests prove that the proposed IEO algorithm has excellent performance.

The remaining structure of this article is as follows: Section 2 introduces the research methods, including the basic Equilibrium Optimizer and the improved Equilibrium Optimizer. In section 3, three function test suites are selected for simulation experiments, such as 23 classical functions and IEEE CEC2017 and IEEE CEC2019. In section 4, the IEO algorithm is applied to six engineering design problems. Finally, the fifth section summarizes the work of this paper.

## 2. Method

In this section, the Equilibrium Optimizer (EO) and the improved Equilibrium Optimizer (IEO) are described in detail respectively. Among them, the improved Equilibrium Optimizer includes three innovation points, and the uniqueness of improved algorithm is reflected by introducing each innovation point.

### 2.1. Equilibrium Optimizer

Equilibrium Optimizer (EO) [18] is a new intelligent algorithm proposed by Faramarzi et al., which is inspired by the mass balance equation in physics. The mass balance equation reflects the physical process of mass entering, leaving and producing in the control volume. In EO, the concentration of each particle is updated in a random way until it reaches equilibrium. EO algorithm constructs three mathematical models: 1. Initialization phase 2. Equilibrium pool and candidates 3. Updating the concentration. The specific description is as follows:

Step1. Initialization phase

Similar to most meta-heuristic algorithms, EO initiates the optimization process by initializing the population. The initial concentration is constructed by randomly initializing the particles in the D - dimensional search space. The initial concentration of each particle is described below:

$$C_{i}^{initial}=Lb+{rand}_{i}(Ub-Lb)\;i=1,2,\ldots ,n$$ (1)

Where $C_{i}^{initial}$ is the initial concentration of the i-th particle, *Ub* and *Lb* represent the maximum and minimum values of particles in the search space, *rand*_*i*_ is a random vector in the range of [0,1], and *n* represents the number of particles.

Step2. Equilibrium pool and candidates (${\vec{C}}_{eq}$)

In order to improve the global search ability of the algorithm and avoid falling into the local optimal solution of low quality, after the initialization phase is completed, the concentration of the generated particles is evaluated and the four particles with the highest fitness value are selected to prepare for the formation of the equilibrium pool.

The equilibrium pool is used to provide candidate solutions during the algorithm optimization process. It consists of four particles with optimal fitness values and one average particle generated during the initialization phase. The mathematical definition is as follows:

$${\vec{C}}_{eq\_ave}=\frac{{\vec{C}}_{eq1}+{\vec{C}}_{eq2}+{\vec{C}}_{eq3}+{\vec{C}}_{eq4}}{4}$$ (2)

$${\vec{C}}_{eq,pool}=\{{\vec{C}}_{eq1},{\vec{C}}_{eq2},{\vec{C}}_{eq3},{\vec{C}}_{eq4},{\vec{C}}_{eq\_ave}\}$$ (3)

Among them, ${\vec{C}}_{eq1}∼{\vec{C}}_{eq4}$ represent the four particles with the highest concentration selected after the initialization of the algorithm, ${\vec{C}}_{eq\_ave}$ represents the average particle, and ${\vec{C}}_{eq,pool}$ represents the equilibrium pool. Especially, in the equilibrium pool, the four particles with the highest concentration contribute to the exploration of the algorithm, while the average particle plays an important role in the exploitation phase.

In the iterative process of the algorithm, each particle is selected from the five candidate particles in the equilibrium pool with the same probability, which contributes to the generation of the global optimal solution.

Step3. Updating the concentration

The exponential term $\vec{F}$ is an important indicator to balance the exploration and exploitation capability of EO algorithm. The calculation of F is as follows:

$$\vec{F}=a_{1}sign(\vec{r}-0.5)∙[e^{-\vec{\lambda }t}-1]$$ (4)

Where *a*_1_ is a constant that controls the exploration ability of the algorithm. $sign(\vec{r}-0.5)$ indicates the direction of exploration and exploitation. $\vec{r}$ and $\vec{\lambda }$ represent vectors within the interval of [0,1], *t* is the coefficient updated with the number of iterations, which can be calculated as follows:

$$t={\left(1-\frac{Iter}{Max\_iter}\right)}^{\left(a_{2}-\frac{Iter}{Max\_iter}\right)}$$ (5)

Where *Iter* represents the current iteration number of the algorithm, and *Max*_*iter* represents the maximum iteration number of the algorithm. *a*_2_ is a constant that can control the exploitation ability of the algorithm. According to the experimental data [18], when *a*_1_ = 2 and *a*_2_ = 1, the performance of algorithm EO is the best. In order to improve the exploitation capability of EO, an equally important indicator is generation rate ($\vec{G}$), which is defined as follows:

$$\vec{G}=\vec{GCP}({\vec{C}}_{eq}-\vec{\lambda }\vec{C})∙\vec{F}$$ (6)

$$\vec{GCP}=\left\{ \begin{array}{cc} 0.5r_{1} & r_{2}\geq GP\\ 0 & r_{2}<GP \end{array}\right.$$ (7)

In the formula, $\vec{GCP}$ represents the control parameter vector of generation rate, $\vec{C}$ represents the current particle concentration, *r*_1_ and *r*_2_ are random numbers within the interval [0,1], and *GP* is a constant with value of 0.5. To sum up, after the concentration update phase of EO algorithm, the update formula of each particle is as follows:

$${\vec{C}=\vec{C}}_{eq}+(\vec{C}-{\vec{C}}_{eq})∙\vec{F}+\frac{\vec{G}}{\vec{\lambda }V}∙(1-\vec{F})$$ (8)

Where *V* is considered as unit.

According to the above description, the updating rule of algorithm EO is to construct the initial concentration of each particle in the initialization phase, select four particles with the highest concentration and form an equilibrium pool with an average particle, which provides candidate solutions for algorithm iteration. Then, the concentration of each particle is calculated using two important indexes: exponential term ($\vec{F}$) and generation rate ($\vec{G}$).

### 2.2. The improved Equilibrium Optimizer

In this paper, an improved Equilibrium Optimizer (IEO) algorithm is proposed. In IEO, there are three improved strategies: Firstly, the algorithm is initialized by using Tent chaotic map instead of randomly generating initial population. The uniform initial population generated by Tent mapping improves the quality of final optimization solution. Second, nonlinear dynamic control parameter is introduced to maintain the balance between the exploration and exploitation phases of the algorithm. Third, Lens Opposition-based Learning (LOBL) is used to calculate the relative population of each iteration process to expand the search space of the algorithm and improve the accuracy of the solution.

#### 2.2.1. Tent chaotic sequence initialization

In the initialization phase, the basic EO algorithm uses the method of random generation to determine the initial solution, which cannot guarantee that the randomly generated initial solution is evenly distributed in the search space. Therefore, in order to improve the quality of the initial solution, Tent chaotic map is introduced [22]. The mathematical expression is as follows:

$$x_{i+1}=\left\{ \begin{array}{l} \frac{x_{i}}{0.7}\;x_{i}<0.7\\ \frac{10}{3}(1-x_{i})\;x_{i}\geq 0.7 \end{array}\right.$$ (9)

Where x_*i*_ shows the chaos variable of i-th particle, *i*∈[1,n].

Tent chaotic mapping has a rich dynamic space, which is a nonlinear phenomenon between determinism and randomness, and has neither periodicity nor convergence. The randomness and ergodic characteristics of Tent chaotic mapping enable the search individual to experience all states without repetition. In EO algorithm, Tent chaotic mapping is introduced to disperse the population as much as possible in the initialization phase, so as to maintain the diversity of the population and improve the global search ability of the algorithm.

In IEO, the Tent chaotic map is used to replace the random distribution to increase the diversity of the population and accelerate the convergence rate of the algorithm. The number of particles is set as *n* and the dimension is set as *d*. The basic steps of initializing particles by using Tent chaotic map within the search range are as follows:

Step1: In the search range, the number of particles is set as *n*, and a group of 1×d vectors are randomly generated, which are taken as the position information of the first particle.

Step2: Using Eq (9), the position information of the remaining *n-1* particles is calculated to form a chaotic sequence.

Step3: The resulting chaotic sequence is initialized by the Eq (10).


$$C_{i}^{initial}=Lb+x_{i}(Ub-Lb)\;i=1,2,\ldots ,n$$ (10)

**Fig 1:**
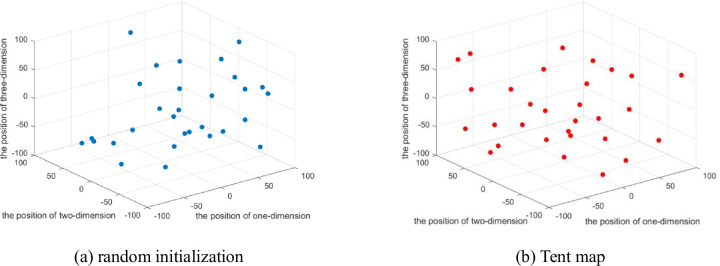
The population distribution of the Sphere function.

**Fig 2:**
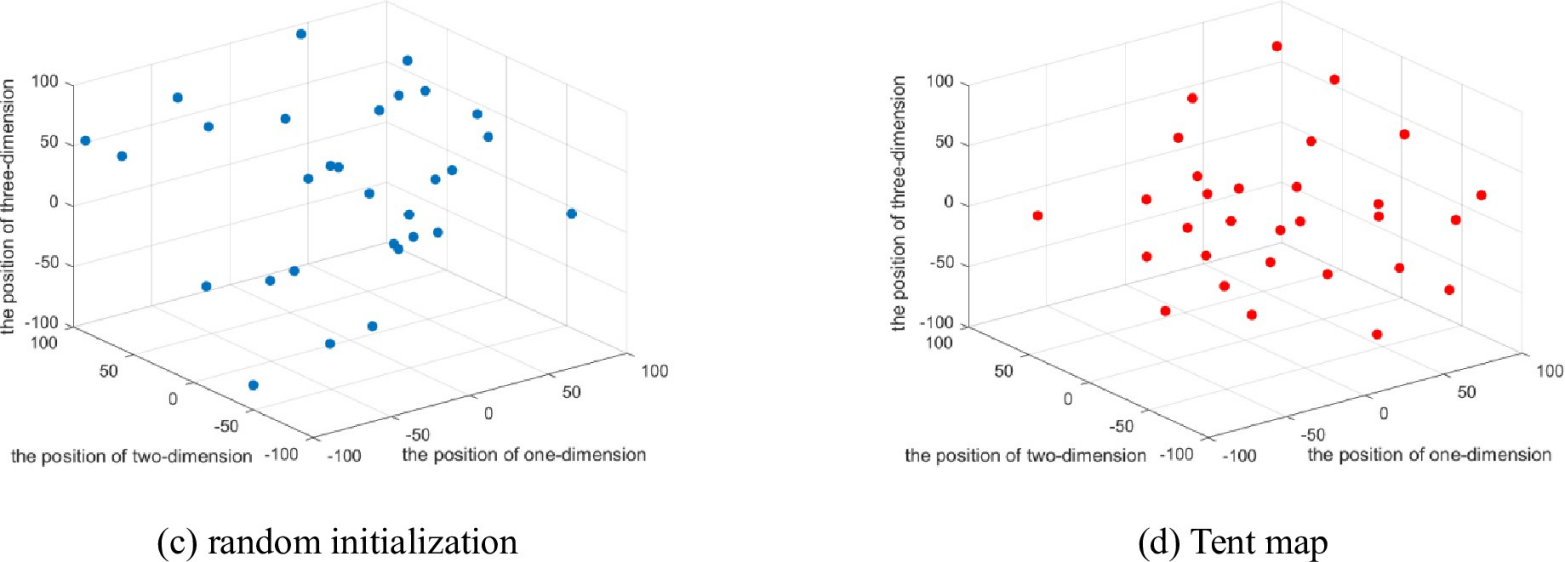
The population distribution of the CEC2017 F15.

In order to verify the rationality of this method, the Tent mapping chaotic sequence is compared with the random initialization in EO algorithm, the particle number is set to 30, and Sphere function among 23 classical reference functions and F15 function in CEC2017 are taken as examples. The details are shown in Figs 1 and 2.

Each point in the figure represents a search individual. As shown in Fig 1, in the search space [–100,100] where Sphere function is located, compared with the population generated by random initialization in Figure (a), the initial population generated by Tent chaotic mapping in Figure (b) is more evenly distributed within the search space. In Fig 2, the F15 function in the CEC2017 test suite is selected for the experiment. Within the search range of [–100,100], it can be clearly seen that the population initialized through the Tent mapping chaotic sequence has a relatively uniform distribution. This improves IEO’s global search capabilities.

#### 2.2.2. Dynamic parameter strategy

In EO, the exponential term $\vec{F}$ is an important index that balances the exploration and exploitation capability of EO algorithm. According to Eq (4), it can be seen that the exponential term $\vec{F}$ is affected by the time parameter *t*. In addition, According to Eq (5), the expression of the time parameter *t* contains the constant *a*_2_, so the change of the parameter *t* largely determines the performance of EO algorithm. In EO, the time parameter *t* decreases from 1 to 0, which is a process that produces nonlinear changes as the number of iterations increases.

According to literature [1], the parameter *t* is redefined and expressed in a new way, as follows:

$$\theta =\frac{\pi }{2}∙\frac{Iter}{Max\_iter}$$ (11)

$$t=t_{end}+(t_{start}-t_{end}){\left(1-sin\theta \right)}^{\left(a_{2}\frac{Iter}{Max\_iter}\right)}$$ (12)

**Fig 3:**
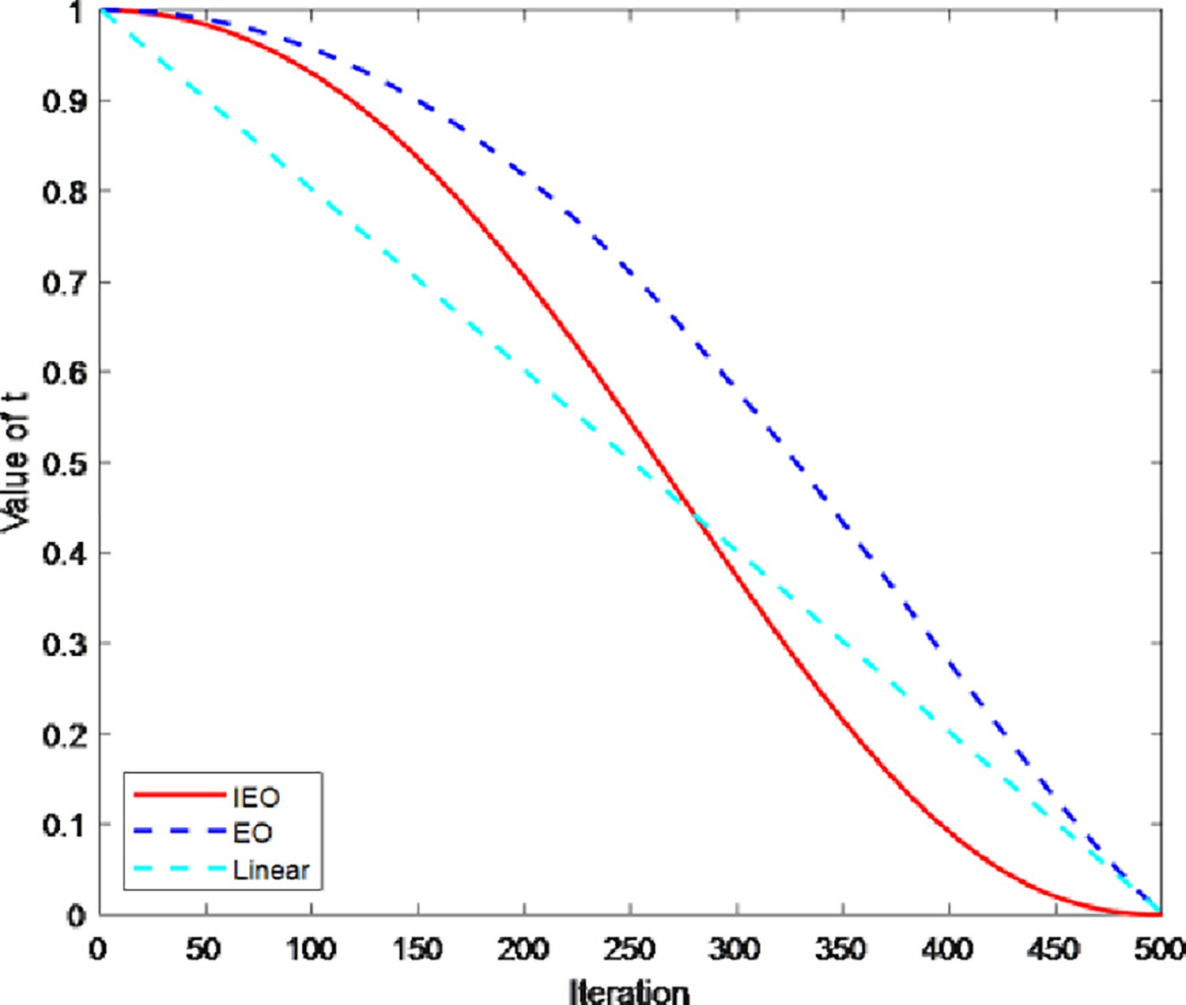
Comparison of parameters.

Among them, *t*_*start*_ = 1; *t*_*end*_ = 0. *Iter* indicates the number of iterations, *Max*_*iter* represents the maximum number of iterations of the algorithm. The change curve of time parameter *t* in nonlinear dynamic parameter strategy, original EO algorithm [18], and linear decline strategy [23] is shown in Fig 3. The dynamic control parameter strategy proposed in this paper reduces slowly in the early stage of algorithm iteration, which avoids premature convergence when the particle is updated, and makes the particle fully search globally in the search space. In addition, in the late iteration of the algorithm, the decreasing speed of parameter *t* is slowed down, so that the particles can search accurately in the search space, thereby the balanced state can be reached more effectively.

#### 2.2.3. Lens Opposition‑based Learning

The original EO algorithm often appears the phenomenon of population aggregation in the late iteration, which makes the algorithm fall into the local extreme value due to the lack of population diversity. In order to strengthen the global search ability of the algorithm and improve the solving accuracy, Lens Opposition Based Learning strategy (LOBL) is applied to EO algorithm. LOBL is used to calculate the opposite solution of candidate solutions in the optimization process of the algorithm. By expanding the opposite region of candidate solutions, the population diversity of the algorithm in the iterative process is enhanced.

**Fig 4:**
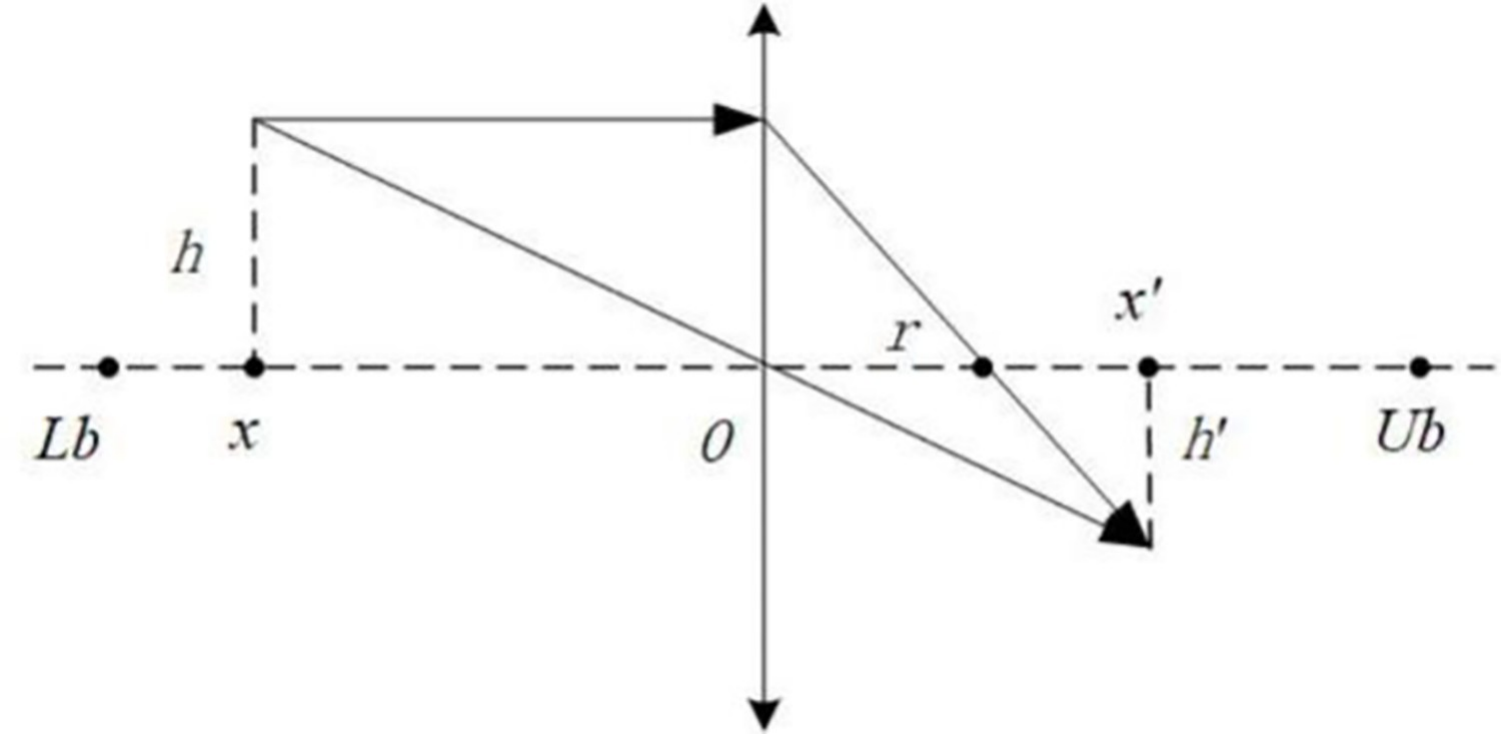
Lens Opposition-based Learning.

The LOBL strategy is a combination of Opposition-based Learning (OBL) strategy and lens imaging principle [24]. When the distance between the object and the convex lens is set to be more than two focal lengths, the process of particles searching for opposite solutions in the search space can be regarded as the process of lens imaging, as shown in Fig 4.

In Fig 4, a convex lens of focal length *r* is placed on the origin *O* (this paper takes (*Ub*+*Lb*)/2). An object of height *h* is placed *x* away from the point *O* and *x* is two focal lengths away. By the lens imaging principle, an image of height *h*′ is generated at point *x*′ on the other side. In other words, the point *x* takes *O* as the basis point to obtain the corresponding reverse point *x*′, and the mathematical relationship is described as follows:

$$\frac{\left(Lb+Ub\right)/2-x}{x^{\prime }-\left(Lb+Ub\right)/2}=\frac{h}{h^{\prime }}$$ (13)

In the above equation, $\frac{h}{h^{\prime }}=k$, and the scaling factor *k* represents the scaling relationship between the object and the corresponding real image. Therefore, the Eq (13) can be transformed into the formula to calculate the opposite solution of *x*′:

$$x^{\prime }=\frac{Lb+Ub}{2}+\frac{Lb+Ub}{2k}-\frac{x}{k}$$ (14)

Since the above equation is only applicable to the opposite solution in one dimensional space, when the optimization problem is multi-dimensional, the solution equation of LOBL strategy is as follows:

$$x_{i}^{\prime }=\frac{{Lb}_{i}+{Ub}_{i}}{2}+\frac{{Lb}_{i}+{Ub}_{i}}{2k}-\frac{x_{i}}{k}$$ (15)

Where $x_{i}^{\prime }$ represents the opposite solution generated by LOBL strategy in the *i-*th dimension, and *Lb*_*i*_ and *Ub*_*i*_ respectively represent the lower bound and upper bound of the *i-*th dimension in the search range.

**Fig 5:**
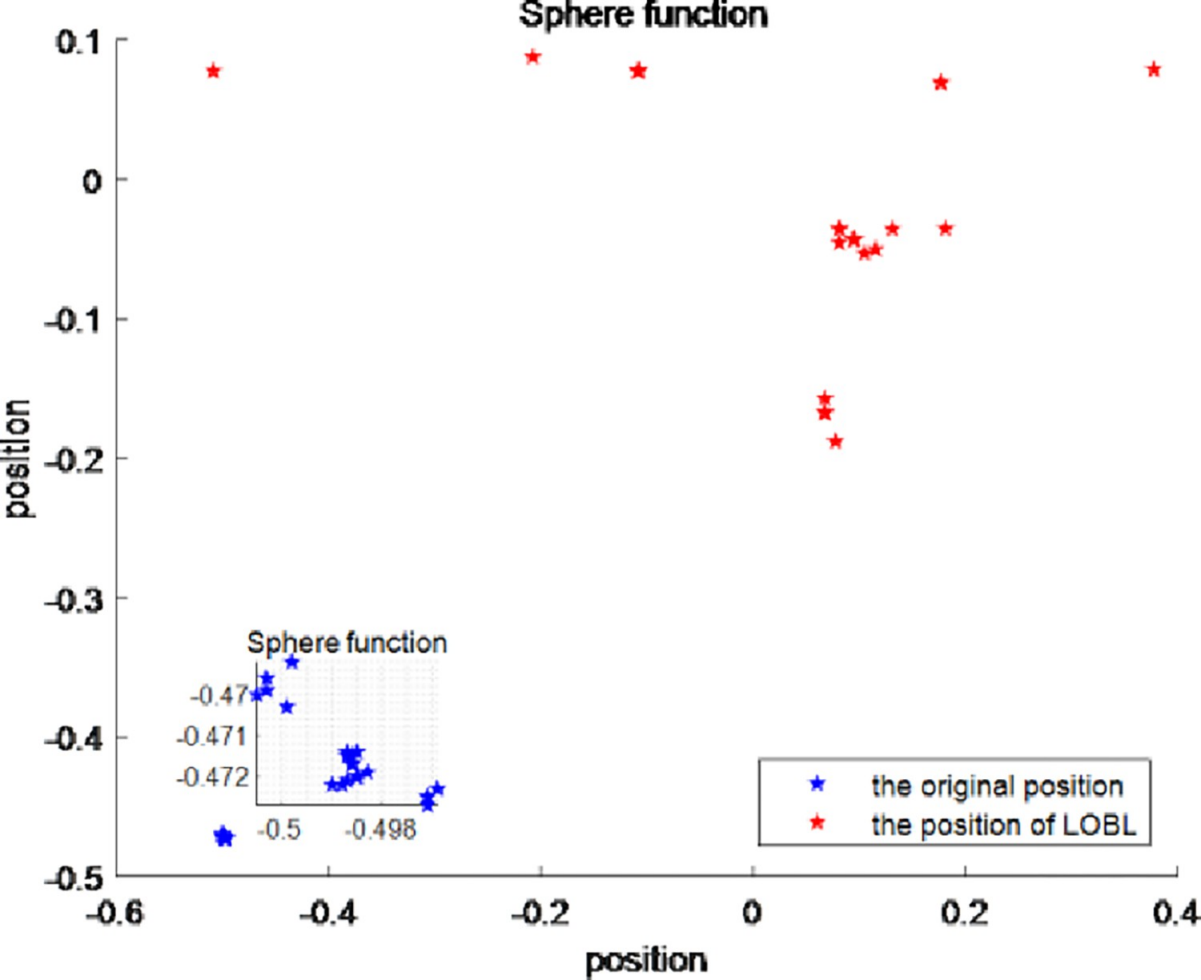
The position of Sphere function generated by LOBL.

**Fig 6:**
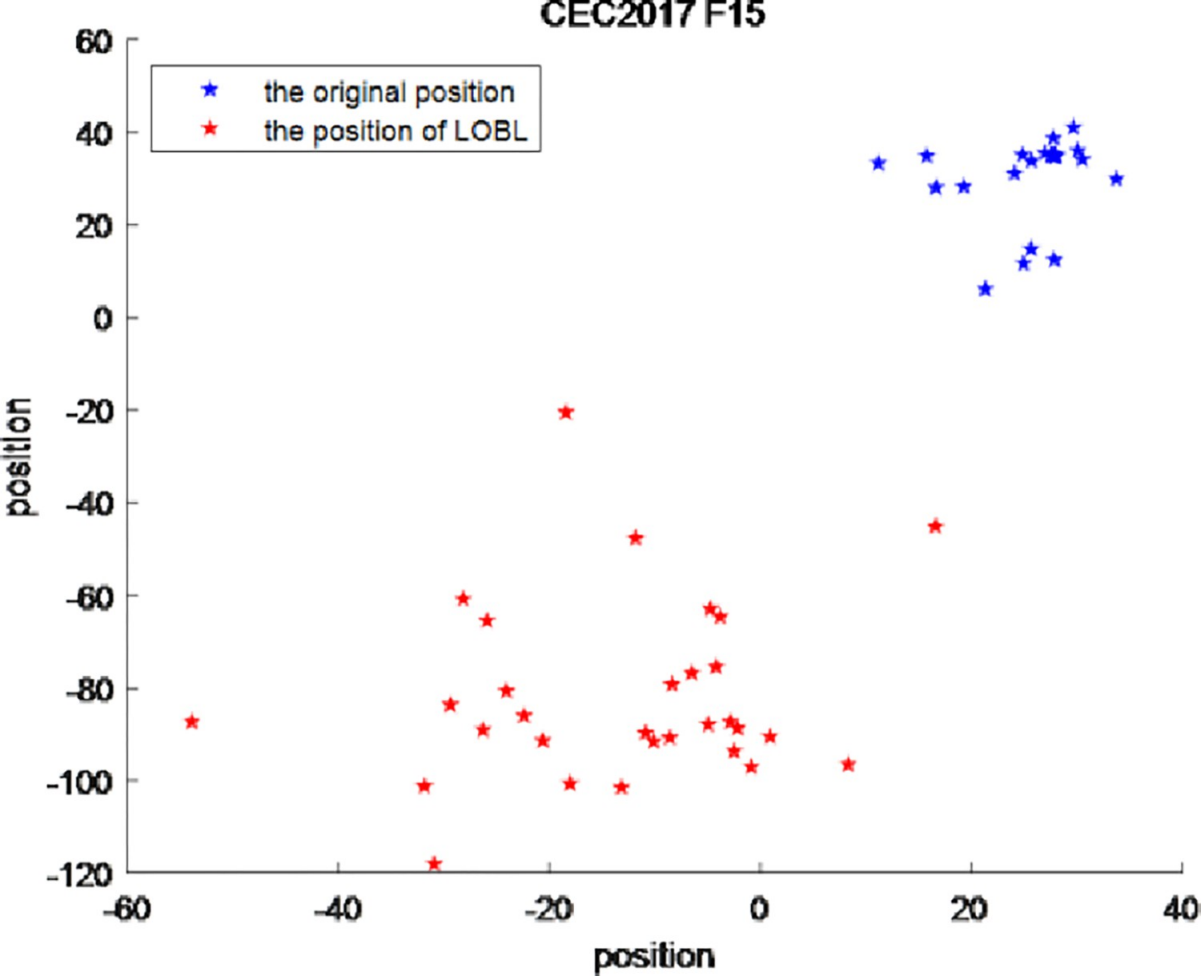
The position of CEC2017 F15 generated by LOBL.

Sphere function among the 23 reference functions and F15 function in the IEEE CEC2017 test suite are taken as examples, and the positions of each particle generated by LOBL strategy are shown in Figs 5 and 6. In the figure, the blue points represent the positions of particles generated by the original EO algorithm when optimizing the function, and the red points represent the positions of particles generated by LOBL strategy. As shown in Fig 5, the original EO algorithm falls into local optimum when optimizing Sphere function, and the positions of each particle produce high overlap. The specific magnified part is shown in the lower left corner of Fig 5. In addition, as can be seen in detail from Fig 6, the original EO algorithm is easy to fall into the plight of local optimal, resulting in a high degree of location overlap of each particle, and the search space becomes increasingly narrow. By introducing LOBL strategy, the opposite solution is generated, which significantly expands the search space of each particle and avoids the algorithm falling into the local optimal solution.

In this paper, the LOBL strategy is used to calculate the opposite solution of the candidate solution generated in each iteration. If the fitness value of the opposite solution is better than the candidate solution, the opposite solution is used to replace the candidate solution to become the current optimal solution, and the iterative operation continues. Therefore, the LOBL strategy significantly improves the ability of particles to escape from the extreme region, which effectively avoids the algorithm falling into the local optimal solution and makes the particles reach the balance state better.

**Fig 7:**
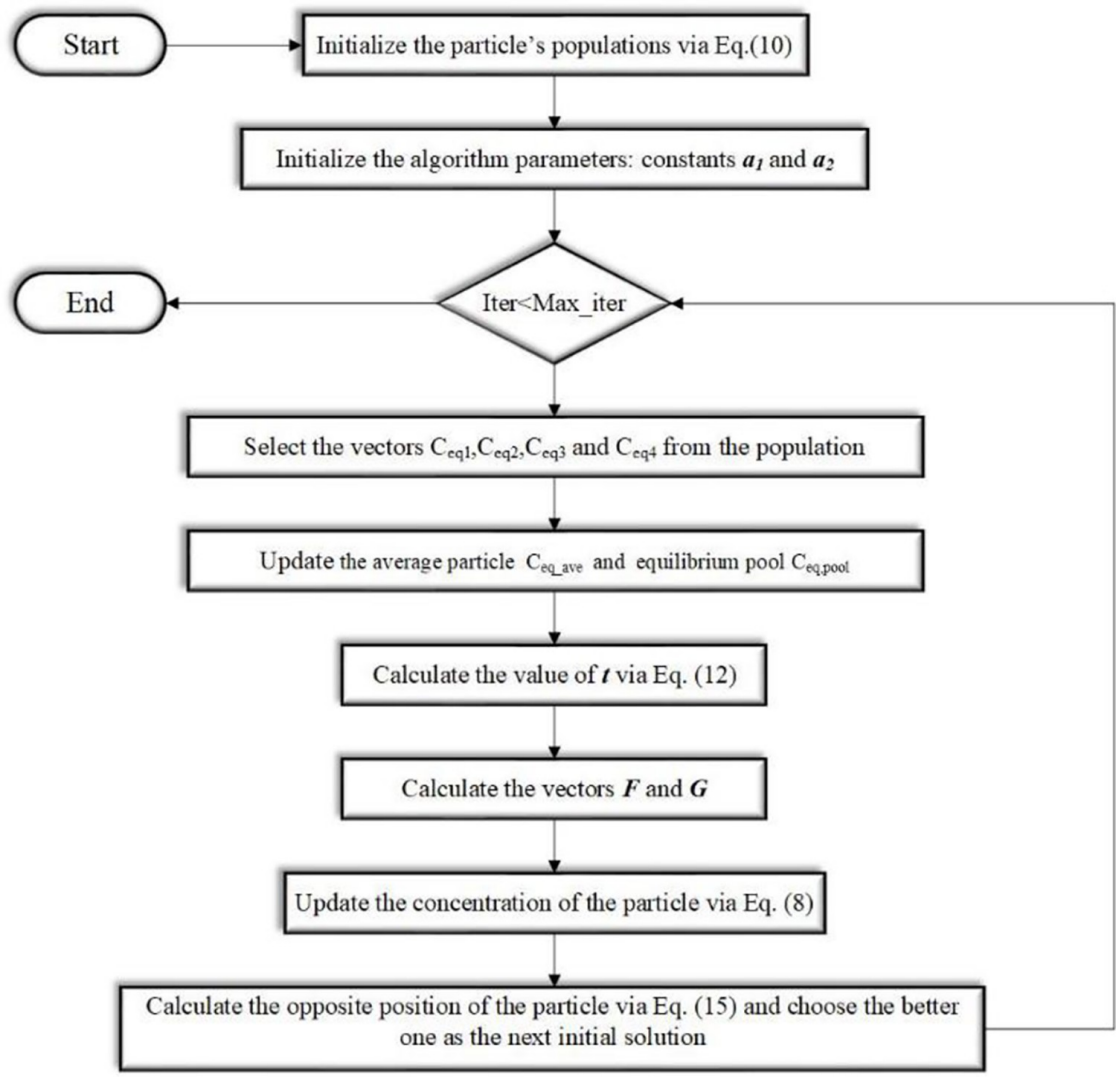
The flowchart of IEO.

In IEO, firstly, the Tent chaotic sequence is used to initialize the particle concentration, so that the initial solution is evenly distributed in the search space as far as possible, and the solving efficiency is improved. Secondly, the new nonlinear dynamic parameter strategy can better balance the exploration and exploitation phases. Finally, the LOBL strategy is used to calculate the opposite solution of the candidate solution generated by each iteration. This strategy avoids falling into the local optimal by increasing the population diversity. These three improved methods can effectively improve the solving speed and accuracy of the algorithm. The pseudocode of the IEO algorithm is illustrated in Algorithm 1. The flow chart of IEO is shown in Fig 7.

Algorithm 1 Improved Equilibrium Optimizer (IEO)

01. Initialize the particle’s populations via Eq (10)

02. Assign equilibrium candidates’ fitness a large number

03. Assign parameter’s value *a*_1_ = 2; *a*_2_ = 1; GP = 0.5

04. **while** (*Iter*<*Max*_*iter*)

05. **for** each particle

06. Calculate fitness of the i-th particle

07. **If *f*(*C***_**1**_**)<***f*(*C*_*eq*1_)

08. Replace *C*_*eq*1_ with *C*_1_ and *f*(*C*_*eq*1_) with *f*(*C*_1_)

09. **elseif *f*(*C***_**1**_**)>***f*(*C*_*eq*1_) and ***f*(*C***_**1**_**)<***f*(*C*_*eq*2_)

10. Replace *C*_*eq*2_ with *C*_1_ and *f*(*C*_*eq*2_) with *f*(*C*_1_)

11. **elseif *f*(*C***_**1**_**)>***f*(*C*_*eq*1_) and ***f*(*C***_**1**_**)>***f*(*C*_*eq*2_) and ***f*(*C***_**1**_**)<***f*(*C*_*eq*3_)

12. Replace *C*_*eq*3_ with *C*_1_ and *f*(*C*_*eq*3_) with *f*(*C*_1_)

13. **elseif *f*(*C***_**1**_**)>***f*(*C*_*eq*1_) and ***f*(*C***_**1**_**)>***f*(*C*_*eq*2_) and ***f*(*C***_**1**_**)>***f*(*C*_*eq*3_) and ***f*(*C***_**1**_**)<***f*(*C*_*eq*4_)

14. Replace *C*_*eq*4_ with *C*_1_ and *f*(*C*_*eq*4_) with *f*(*C*_1_)

15. **end if**

16. **end for**

17. calculate the average particle *C*_*eq*_*ave*_ via Eq (2)

18. construct the equilibrium pool *C*_*eq*,*pool*_ via Eq (3)

19. Implement the memory saving

20. Calculate the value of *t* via Eq (12)

21. **for** each particle

22. Choose a random candidate from the concentration pool

23. Calculate the values of vectors *F* and *G* via Eq (4), Eq (6) and Eq (7)

24. Update the concentration of the particle via Eq (8)

25. Calculate the opposite position of the particle via Eq (15)

26. Choose the better one as the next initial solution

27. **end for**

28. *Iter* = *Iter* +1

29. **end while**

#### 2.2.4. Computational complexity

Time complexity is one of the criteria for checking algorithm performance. In this article, big-O notation is used to represent complexity [1]. The computational complexity of the algorithm includes three main parts: initialization, fitness evaluation and population updating mechanism. The complexity calculations for the original EO and the improved IEO are as follows.

The original EO initializes the concentration of each particle in O(N×D) time, where N represents the number of particles and D represents the dimension of the problem. The fitness assessment of each particle requires O(N) time. And the selection of the particle with the highest concentration requires O(N) time. The concentration of update mechanism in the original EO requires O(N×D) time. Thus for the total Max_-_iter iterations, the total computation complexity of the original EO is equivalent to O(N×D×Max_-_iter).

The initialization of particle concentration in IEO requires O(N×D) time, where N represents the number of particles and D represents the dimension of the problem. The fitness assessment of each particle requires O(N) time. Moreover, the selection of the particle with the highest concentration requires O(N) time. The concentration of update mechanism in the IEO requires O(N×D) time. Tent chaos strategy takes O (N) time. And the Lens Opposition-based Learning strategy requires O (N) time. Thus for the total Max_-_iter iterations, the total calculation complexity of IEO is equivalent to O(N×D×Max_-_iter). Consequently, the original EO and the proposed IEO are identical in terms of time complexity.

## 3. Numerical experiment results and analysis

In this section, 23 classical benchmark functions and two test suite functions IEEE CEC2017 and IEEE CEC2019 with complex changes are selected to carry out simulation experiments. The CEC2017 and CEC2019 test suites contain different functions, which are divided into different categories: basic functions, hybrid functions, and composition functions. Among them, the CEC2017 data suite includes 30 complex composite functions, and the CEC2019 test suite includes 10 functions. These functions have different rotation matrices, each matrix is generated from standard normally distributed entries by Gram-Schmidt ortho-normalization with condition number *c* that is equal to 1 or 2. Therefore, these functions have stable performance in numerical experiments, which can show the optimization performance of the test algorithm.

In the experiment, the performance of IEO optimization results is analyzed and compared with six famous meta-heuristic algorithms, which are Equilibrium Optimizer (EO) [18], salp swarm algorithm (SSA) [25], sine and cosine algorithm (SCA) [23], butterfly optimization algorithm (BOA) [26], the particle swarm optimization (PSO) [14] and bat algorithm (BA) [27]. Among them, PSO and BA are classical swarm intelligence algorithms. In recent years, these two algorithms are not only widely used in algorithm optimization [28], but also applied to hot fields such as neural network [29] and artificial intelligence [30]. SSA, SCA and BOA algorithms are representative intelligent algorithms emerging in recent years. These three algorithms have good global optimization ability and are widely used in function optimization problems [31]. The experimental parameter values of all algorithms are shown in Table 1, which are selected from the original paper of each algorithm.

**Table 1:** The parameters of the algorithms.

Algorithm	Parameters	Values
EO	Constant coefficient (**a**_**1**_)	2
	Constant coefficient (***a***_***2***_)	1
SCA	Convergence factor (***r***_***1***_)	[0,2]
	Constant coefficient (***a***)	2
SSA	Constant coefficient (***c***_***1***_)	[2/e,2]
PSO	Maximum Inertia weight (***Wmax***)	0.09
	Minimum Inertia weight (***Wmin***)	0.04
	Minimum Velocity (***Vmin***)	-5
	Maximum Velocity (***Vmax***)	5
	Constant coefficient (***c***_***1***_)	2
	Constant coefficient (***c***_***2***_)	2
BOA	Modular modality (***c***)	0.01
	Power exponent (***a***)	[0.1,0.3]
BA	Frequency minimum (***Qmin***)	0
	Frequency maximum (***Qmax***)	2
	Loudness (***A***)	0.5
	Pulse rate (***r***)	0.5

For fair comparison, three standards of mean value (mean), standard deviation (std) and running time (time) are used as the evaluation indexes. The average value intuitively shows the results of function optimization of each algorithm, and the standard deviation reflects the dispersion degree of optimization data. The smaller the standard deviation is, the higher the stability of the algorithm is. And the running time directly shows the convergence speed of the algorithm. The following three experiments describe in detail the optimization of the improved algorithm IEO for different problems.

### 3.1. 23 classical benchmark functions

In this section, 23 classical benchmark functions are selected for simulation experiments [17]. These functions are divided into unimodal functions and multimodal functions. Among them, F1-F7 is a unimodal function with only one global optimal solution, which is mainly used to test the optimization accuracy of the algorithm. The multimodal functions F8-F23 has multiple optima and is easy to fall into local optima. It is often used to test the exploration ability of the algorithm and the ability to avoid local optima. In addition, F14-F23 belong to fixed dimensional multimodal functions, whose dimensions are lower and fixed, so they have fewer local optimal solutions.

In order to ensure the fairness and comparability of the experiment, this paper sets the population number of the seven algorithms as 30, the maximum number of iterations as 500, and the dimension of the algorithms as 30. Each algorithm runs the test function for 30 times independently and records the mean value (mean), standard deviation (std) and running time (time) of the experimental data. The optimization results of all the algorithms for the 23 classical functions are shown in Table 2. Note that the bold values in the table represent the best results for each function optimization.

**Table 2:** The comparison results of different algorithms on 23 benchmark functions with D=30.

Function	Result	IEO	EO	SSA	SCA	BOA	PSO	BA
F1	Mean	**0**	2.85E-41	1.48E-07	10.8237	1.31E-11	2.70E-73	3.34E+03
	Std	**0**	5.08E-41	1.25E-07	11.3405	1.14E-12	1.43E-72	5.47E+03
	Time	6.851 s	**3.921 s**	5.330 s	7.124 s	4.276 s	9.031 s	6.886 s
F2	Mean	**1.08E-166**	8.08E-24	2.2539	0.0207	4.32E-09	1.48E-51	33.8185
	Std	**0**	7.82E-24	2.0791	0.0378	1.40E-09	5.78E-51	23.4872
	Time	**4.434 s**	4.689 s	5.164 s	7.234 s	4.977 s	6.436 s	7.239 s
F3	Mean	**2.01E-315**	1.27E-09	1.78E+03	6.87E+03	1.29E-11	4.31E+04	1.91E+04
	Std	**0**	3.54E-09	1.03E+03	4.15E+03	1.12E-12	1.41E+04	8.99E+03
	Time	**6.132 s**	8.046 s	9.667 s	13.303 s	13.114 s	10.138 s	11.250 s
F4	Mean	**8.21E-158**	3.46E-10	11.7559	34.3367	5.99E-09	44.3426	70.3417
	Std	**1.39E-157**	7.56E-10	3.0076	11.9158	4.42E-10	26.9932	8.7013
	Time	**4.027 s**	5.924 s	5.212 s	7.107 s	4.537 s	5.721s	6.966 s
F5	Mean	25.4623	**25.3233**	281.2543	1.09E+05	28.9402	27.9294	2.68E+06
	Std	0.2692	0.2454	783.4751	2.60E+05	**0.0321**	0.428	1.46E+07
	Time	**5.133 s**	5.388 s	5.695 s	9.055 s	5.451 s	6.427 s	7.729 s
F6	Mean	3.67E-06	7.58E-06	**1.53E-07**	17.3566	6.0152	0.3729	3.01E+03
	Std	2.82E-06	7.00E-06	**2.15E-07**	23.2015	0.5234	0.2593	5.35E+03
	Time	**4.135 s**	4.686 s	5.724 s	7.325 s	4.251 s	6.875 s	7.350 s
F7	Mean	**0**	1.29E-74	2.95E-11	0.0229	1.15E-14	1.51E-111	4.3848
	Std	**0**	3.02E-74	7.31E-11	0.0437	1.33E-15	8.28E-111	12.7958
	Time	8.217 s	8.071 s	**7.565 s**	9.468 s	8.687 s	8.396 s	9.114 s
F8	Mean	-8.82E+03	-8.96E+03	-7.62E+03	-3.77E+03	-3.72E+03	**-9.97E+03**	-8.33E+03
	Std	537.6766	582.6549	729.6419	350.8453	351.4027	**1.70E+03**	792.1073
	Time	**5.740 s**	7.133 s	5.763 s	7.699 s	9.702 s	9.434 s	7.624 s
F9	Mean	**0**	**0**	50.0795	49.4458	2.93E-09	5.68E-15	168.2969
	Std	**0**	**0**	13.9522	39.0467	1.37E-08	2.29E-14	34.0651
	Time	**5.323 s**	6.050 s	5.443 s	7.499 s	6.120 s	8.631 s	7.643 s
F10	Mean	**2.31E-15**	8.35E-15	2.8441	15.8001	6.04E-09	5.15E-15	15.8891
	Std	**1.77E-15**	2.16E-15	1.1462	7.9825	5.53E-10	2.70E-15	6.2066
	Time	**4.760 s**	5.098 s	5.639 s	9.342 s	5.335 s	7.563 s	7.442 s
F11	Mean	**0**	6.56E-04	0.0169	0.9049	4.65E-12	0.0054	27.9399
	Std	**0**	0.0036	0.0126	0.3775	2.55E-12	0.0294	53.6106
	Time	**4.435 s**	4.631 s	5.953 s	8.234 s	5.522 s	6.963 s	7.974 s
F12	Mean	**5.93E-08**	4.70E-07	7.7091	6.24E+04	0.6566	0.0228	12.0731
	Std	**9.98E-08**	4.08E-07	4.1368	2.00E+05	0.1838	0.0138	21.5277
	Time	**12.365 s**	13.563 s	12.831 s	14.402 s	17.617 s	19.845 s	13.707 s
F13	Mean	0.6807	**0.0192**	17.3181	5.79E+05	2.9499	0.5275	1.01E+03
	Std	1.0265	**0.0371**	15.2915	2.27E+06	0.1343	0.2578	4.96E+03
	Time	16.196 s	**10.569 s**	12.039 s	13.950 s	17.743 s	20.849 s	14.132 s
F14	Mean	1.1964	**0.998**	1.1304	1.7264	1.5197	3.5412	3.3945
	Std	0.6054	**1.37E-16**	0.431	0.9718	0.6952	4.099	2.8086
	Time	19.607 s	15.460 s	14.522 s	**13.667 s**	27.643 s	14.158 s	14.748 s
F15	Mean	**3.54E-04**	0.0011	0.0028	0.0011	4.03E-04	6.95E-04	0.0017
	Std	**1.72E-04**	0.0037	0.006	3.51E-04	1.29E-04	4.69E-04	0.0036
	Time	3.841 s	3.733 s	3.261 s	**2.600 s**	4.372 s	3.504 s	2.790 s
F16	Mean	**-1.0316**	-1.0316	-1.0316	-1.0316	-9.94E+02	-1.0316	-1.0316
	Std	6.12E-16	6.12E-16	3.38E-14	4.38E-05	3.08E+03	1.02E-09	**6.78E-16**
	Time	3.725 s	3.626 s	3.278 s	**2.203 s**	6.024 s	2.806 s	2.418 s
F17	Mean	**0.3979**	0.3979	NAN	0.3997	NAN	0.3979	0.3979
	Std	**0**	0	NAN	0.0032	NAN	2.96E-05	0
	Time	**2.362 s**	3.398 s	NAN	2.190 s	NAN	2.530 s	2.642 s
F18	Mean	**3**	3	3	3.0001	3.3123	3	3
	Std	1.44E-15	**1.33E-15**	2.87E-13	1.33E-04	1.109	1.09E-04	2.00E-15
	Time	3.273 s	3.047 s	2.821 s	**2.008 s**	4.035 s	2.576 s	2.269 s
F19	Mean	**-3.8628**	-3.8628	-3.8628	-3.8539	-4.0035	-3.8565	-3.8628
	Std	**2.49E-15**	2.57E-15	1.81E-11	0.0028	0.3408	0.0086	2.71E-15
	Time	**2.845 s**	3.013 s	4.302 s	3.212 s	9.666 s	3.379 s	2.926 s
F20	Mean	-3.8625	**-3.8628**	-3.8628	-3.8536	-3.7484	-3.8528	-3.8628
	Std	0.0014	**2.54E-15**	8.20E-11	0.0026	3.6326	0.0202	2.71E-15
	Time	5.004 s	3.968 s	3.924 s	4.229 s	9.929 s	4.655 s	**3.648 s**
F21	Mean	-8.5634	**-8.8053**	-7.3911	-2.5161	-4.4844	-8.5282	-5.7239
	Std	2.5078	2.5419	3.3142	1.7151	**0.3622**	2.492	3.3265
	Time	**4.125 s**	5.649 s	4.270 s	4.442 s	10.798 s	4.304 s	5.954 s
F22	Mean	-9.4631	**-10.0031**	-8.6979	-3.6573	-4.2444	-8.0746	-7.5472
	Std	2.4778	1.532	3.1699	1.6085	**0.6247**	3.1563	3.4071
	Time	6.265 s	4.699 s	4.372 s	**3.602 s**	12.840 s	4.561 s	4.003 s
F23	Mean	**-9.9204**	-9.8623	-8.9919	-4.0564	-4.0375	-6.8564	-7.2819
	Std	1.9106	2.0874	2.9026	2.0752	**0.553**	3.2605	3.8045
	Time	5.304 s	5.270 s	**4.802 s**	5.235 s	16.033 s	5.340 s	4.917 s
Average rank		**2.18**	2.25	4.23	5.82	4.18	3.70	5.64
Rank		**1**	2	5	7	4	3	6

As can be seen from Table 2, IEO improves accuracy by several orders of magnitude for most benchmark functions. For unimodal function F1-F7, the optimization efficiency of the algorithm IEO is greatly improved, which indicates that IEO has strong exploitation ability and can find the global optimal solution. For multimodal functions F8-F23, the average value of fitness obtained by IEO in the optimization process of ten functions F9-F12, F15-F19 and F23 is optimal. This shows that the improved IEO has strong exploration ability and robustness when dealing with multimodal functions. In general, three improvement strategies enhance the optimization performance of the algorithm and accelerates convergence speed. The improved Equilibrium Optimizer achieves complementary advantages to enhance the global search ability so that the IEO can find an accurate solution.

The convergence speed of the algorithm is reflected by calculating the running time of each function. For the 30-dimensional functions F1-F13, except for functions F1, F7 and F13, the running time of IEO algorithm is relatively fast. That is to say, IEO has a good convergence speed when solving unimodal and multimodal functions. For low dimensional multimodal functions F14-F23, these functions belong to 2-dimensional, 4-dimensional and 6-dimensional functions, and the optimization effect of IEO is not outstanding. In general, the functions F1-F13 are tested on the basis of 30 dimensions, and IEO can improve the solution accuracy and avoid local optima when dealing with high-dimensional functions. However, for low-dimensional functions F14-F23, the IEO algorithm tends to fall into the local optimum when solving lower-dimensional functions, which greatly reduces the convergence speed and solution efficiency of the algorithm.

**Fig 8:**
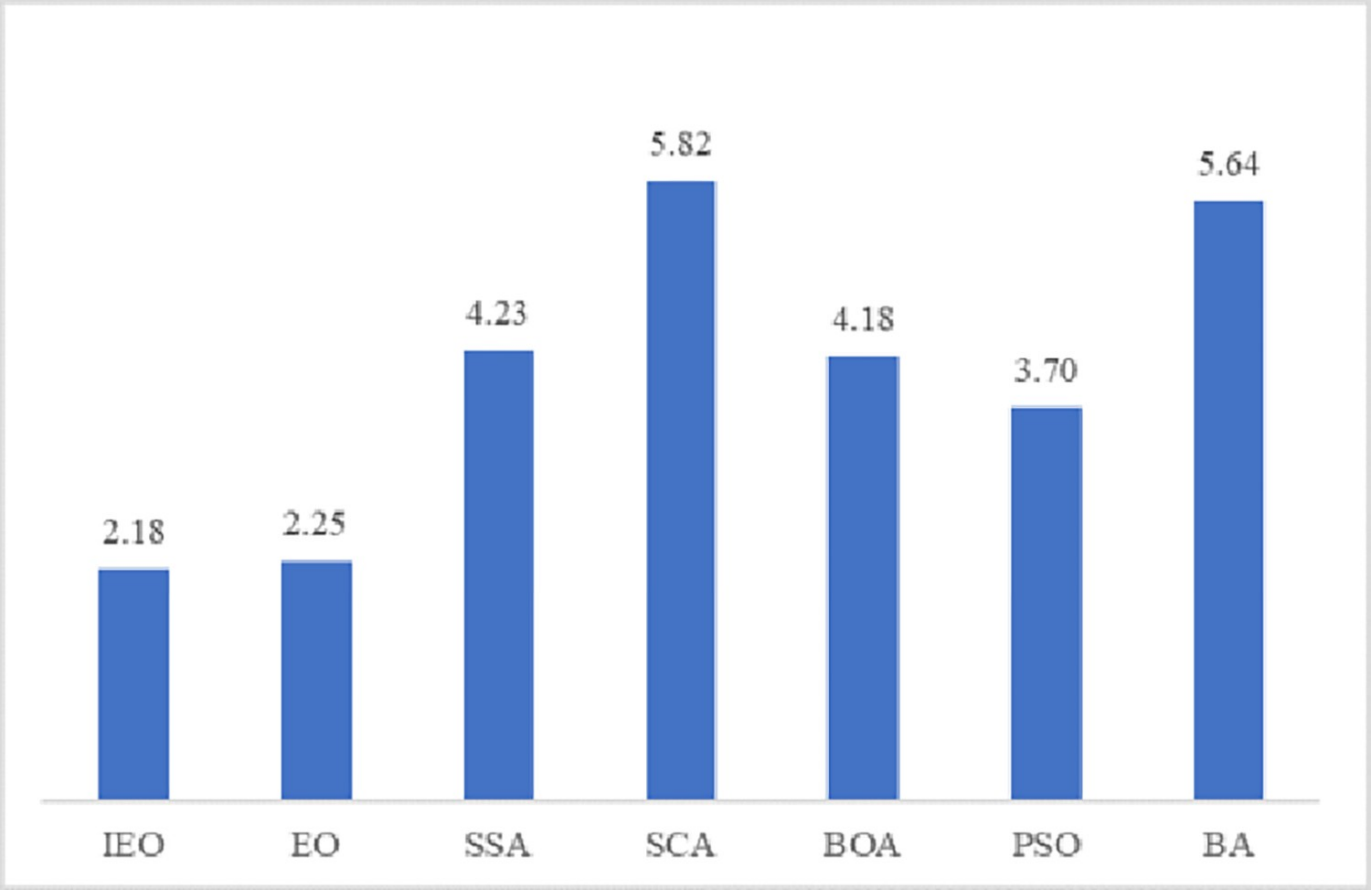
Mean rank of Friedman test on 23 benchmark functions.

In order to analyze the optimization results of IEO and other algorithms more effectively, the Friedman test is selected as a further evaluation index in the experiment. The order is based on the mean and standard deviation of all the algorithms. If the mean is smaller, the rank obtained by the Friedman test is smaller. In the experiment, the software IBM SPSS24 is used to calculate each algorithm, and the sorting results are shown in Table 2. IEO’s average rank is 2.18, ranking first among the seven algorithms. In addition, in order to view the average rank results of each algorithm more clearly, the Fig 8 is drawn, which shows that the ranking of IEO is better than other algorithms. The results of Friedman simulation experiment show that IEO has superior performance compared with the original EO algorithm and PSO. The improved Equilibrium Optimizer not only has strong robustness and stability but also has a faster convergence speed and higher calculation accuracy.

## 3.2. IEEE CEC2017 functions

The conditions of the CEC2017 test suite are more complex and challenging than unconstrained functions. Therefore, the CEC2017 is selected as the optimization problem to evaluate the algorithm IEO. For the CEC2017 test suite [32], there are four types of problems: they are: F1-F3 are unimodal rotation displacement functions, which are usually used to evaluate the convergence rate and optimization accuracy of the algorithm; F4-F10 are multimodal rotation displacement functions, which are usually used to reflect the ability of the algorithm to avoid local optimality. In addition, F11-F20 are hybrid functions, and F21-F30 are composition functions. It is difficult for most algorithms to reach the global optimal solution of the hybrid functions and the composite functions. Among them, F2 is deleted from the function list due to its instability and is not studied. Therefore, this section conducts simulation experiments on the basis of 29 functions, and the specific information is shown in Table 3.

**Table 3:** Description of CEC2017 benchmark functions.

ID	Functions	Class	Optimum
F1	Shifted and rotated bent cigar function	Unimodal	100
F2	Shifted and rotated sum of different power function	Unimodal	200
F3	Shifted and rotated Zakharov function	Unimodal	300
F4	Shifted and rotated Rosenbrock’s function	Multimodal	400
F5	Shifted and rotated Rastrigin’s function	Multimodal	500
F6	Shifted and rotated expanded Scaffer’s F6 function	Multimodal	600
F7	Shifted and rotated Lunacek bi-Rastrigin function	Multimodal	700
F8	Shifted and rotated non-continuous Rastrigin’s function	Multimodal	800
F9	Shifted and rotated Levy function	Multimodal	900
F10	Shifted and rotated Schwefel’s function	Multimodal	1000
F11	Hybrid function 1 (N=3)	Hybrid	1100
F12	Hybrid function 2 (N=3)	Hybrid	1200
F13	Hybrid function 3 (N=3)	Hybrid	1300
F14	Hybrid function 4 (N=4)	Hybrid	1400
F15	Hybrid function 5 (N=4)	Hybrid	1500
F16	Hybrid function 6 (N=4)	Hybrid	1600
F17	Hybrid function 6 (N=5)	Hybrid	1700
F18	Hybrid function 6 (N=5)	Hybrid	1800
F19	Hybrid function 6 (N=5)	Hybrid	1900
F20	Hybrid function 6 (N=6)	Hybrid	2000
F21	Composition function 1 (N=3)	Composition	2100
F22	Composition function 2 (N=3)	Composition	2200
F23	Composition function 3 (N=4)	Composition	2300
F24	Composition function 4 (N=4)	Composition	2400
F25	Composition function 5 (N=5)	Composition	2500
F26	Composition function 6 (N=5)	Composition	2600
F27	Composition function 7 (N=6)	Composition	2700
F28	Composition function 8 (N=6)	Composition	2800
F29	Composition function 9 (N=3)	Composition	2900
F30	Composition function 10 (N=3)	Composition	3000

In the experiment, the above six meta-heuristic algorithms are selected for comparison. In order to maintain the fairness of experimental data, the population number is uniformly set as 30, the maximum number of iterations is set as 500, and the dimension of the function is 30. All functions are independently run for 30 times and their average value (mean), standard deviation (std) and running time (time) are recorded. Details of the experimental data are shown in Table 4.

**Table 4:** The comparison results of different algorithms on CEC2017 functions with D=30.

Function	Result	IEO	EO	SSA	SCA	BOA	PSO	BA
F1	Mean	6.64E+04	1.56E+05	**7.67E+03**	2.20E+10	5.61E+10	5.59E+09	1.03E+10
	Std Time	8.37E+04 5.539 s	3.50E+05 5.388 s	**7.19E+03 3.953 s**	3.36E+09 8.554 s	9.76E+09 5.723 s	1.80E+09 7.643 s	6.70E+09 8.293 s
F3	Mean	**4.26E+04**	5.28E+04	7.56E+04	8.20E+04	7.96E+04	2.57E+05	1.55E+05
	Std	**7.34E+03**	1.25E+04	2.69E+04	1.67E+04	9.67E+03	7.02E+04	5.72E+04
	Time	6.329 s	6.118 s	5.935 s	8.450 s	**5.712 s**	7.154 s	8.593 s
F4	Mean	**511.7805**	516.734	534.4998	3.00E+03	2.04E+04	1.37E+03	1.35E+03
	Std	**18.6095**	24.0721	44.3882	6.87E+02	3.59E+03	473.671	844.6995
	Time	5.294 s	5.001 s	5.763 s	**4.437 s**	5.738 s	7.975 s	8.574 s
F5	Mean	**5.99E+02**	603.0197	6.84E+02	831.9749	9.17E+02	860.098	695.3999
	Std	**2.58E+01**	28.7098	4.07E+01	28.8511	2.84E+01	50.5377	45.4718
	Time	**5.829 s**	6.511 s	6.240 s	9.084 s	7.266 s	9.009 s	9.084 s
F6	Mean	609.3797	**6.02E+02**	6.53E+02	661.6059	6.91E+02	682.8361	637.6466
	Std	4.1784	**1.7633**	1.02E+01	7.1982	7.09E+00	12.6851	10.3965
	Time	**9.428 s**	10.243 s	9.523 s	10.982 s	10.352 s	11.095 s	10.669 s
F7	Mean	872.0349	**8.58E+02**	9.79E+02	1.26E+03	1.40E+03	1.31E+03	1.11E+03
	Std	41.1012	**39.3815**	9.64E+01	60.4739	3.57E+01	102.3825	167.919
	Time	**5.975 s**	6.231 s	6.690 s	8.989 s	7.284 s	9.210 s	9.026 s
F8	Mean	896.4204	**8.91E+02**	9.67E+02	1.09E+03	1.14E+03	1.07E+03	1.01E+03
	Std	25.4733	2.66E+01	3.87E+01	24.2593	**22.8111**	61.2718	51.044
	Time	6.907 s	6.156 s	**4.599 s**	8.698 s	7.592 s	8.686 s	8.955 s
F9	Mean	2.65E+03	**1.24E+03**	5.68E+03	9.01E+03	1.12E+04	1.06E+04	7.58E+03
	Std	1.25E+03	**5.37E+02**	1.79E+03	1.88E+03	1.36E+03	3.26E+03	2.40E+03
	Time	6.964 s	6.367 s	**6.341 s**	9.276 s	7.145 s	9.218 s	9.188 s
F10	Mean	**4.73E+03**	5.47E+03	5.51E+03	8.89E+03	9.11E+03	7.77E+03	5.53E+03
	Std	525.528	9.43E+02	7.86E+02	332.9991	**314.9877**	627.4289	653.9668
	Time	**7.654 s**	8.137 s	7.763 s	9.328 s	7.952 s	9.571 s	9.850 s
F11	Mean	**1.24E+03**	1.25E+03	1.41E+03	3.81E+03	8.68E+03	8.83E+03	5.52E+03
	Std	**37.8988**	4.80E+01	116.0291	717.8026	2.24E+03	4.14E+03	4.92E+03
	Time	9.604 s	9.020 s	8.068 s	8.446 s	**6.455 s**	7.919 s	8.714 s
F12	Mean	**1.30E+06**	1.69E+06	4.19E+07	2.68E+09	1.39E+10	5.31E+08	1.99E+08
	Std	**1.04E+06**	1.33E+06	3.69E+07	7.32E+08	4.34E+09	5.00E+08	5.04E+08
	Time	9.908 s	9.284 s	**6.697 s**	8.679 s	7.752 s	8.037 s	8.975 s
F13	Mean	2.27E+04	**2.16E+04**	1.51E+05	1.14E+09	1.07E+10	1.29E+07	4.33E+07
	Std	**1.57E+04**	2.05E+04	7.86E+04	5.33E+08	5.32E+09	9.94E+06	1.93E+08
	Time	6.934 s	6.748 s	**6.408 s**	8.898 s	6.677 s	8.059 s	8.782 s
F14	Mean	**5.22E+04**	6.84E+04	1.34E+05	9.13E+05	4.52E+06	3.18E+06	8.64E+05
	Std	**5.08E+04**	5.52E+04	1.14E+05	5.05E+05	5.40E+06	4.13E+06	2.13E+06
	Time	**8.585 s**	8.864 s	10.156 s	9.094 s	10.817 s	9.715 s	9.596 s
F15	Mean	**4.49E+03**	8.71E+03	6.69E+04	5.54E+07	6.86E+08	7.71E+06	5.42E+04
	Std	**3.41E+03**	9.31E+03	7.24E+04	4.07E+07	4.38E+08	9.29E+06	3.98E+04
	Time	5.608 s	**5.488 s**	6.454 s	8.402 s	6.430 s	9.090 s	8.791 s
F16	Mean	**2.44E+03**	2.61E+03	3.12E+03	4.12E+03	8.73E+03	4.48E+03	3.02E+03
	Std	284.6801	333.867	3.37E+02	**264.0054**	1.87E+03	720.7158	425.317
	Time	**6.768 s**	6.831 s	7.264 s	8.804 s	6.795 s	7.610 s	8.981 s
F17	Mean	**2.07E+03**	2.11E+03	2.35E+03	2.82E+03	1.76E+04	2.86E+03	2.49E+03
	Std	**178.033**	228.2042	228.2704	199.3174	1.74E+04	293.9794	249.1799
	Time	**9.634 s**	10.044 s	11.036 s	10.538 s	10.128 s	11.131 s	10.213 s
F18	Mean	**3.34E+05**	9.27E+05	2.32E+06	1.47E+07	5.89E+07	1.45E+07	1.20E+07
	Std	**3.40E+05**	9.15E+05	2.60E+06	7.99E+06	6.64E+07	1.78E+07	2.09E+07
	Time	6.708 s	**6.409 s**	6.778 s	8.648 s	7.265 s	9.516 s	8.914 s
F19	Mean	**4.96E+03**	8.14E+03	5.06E+06	1.16E+08	9.74E+08	2.13E+07	2.37E+07
	Std	**1.76E+03**	8.26E+03	3.11E+06	6.30E+07	1.19E+09	1.96E+07	5.13E+07
	Time	**14.877 s**	15.915 s	17.233 s	17.659 s	24.749 s	25.116 s	17.894 s
F20	Mean	2.45E+03	**2.42E+03**	2.59E+03	2.92E+03	3.06E+03	2.94E+03	2.71E+03
	Std	180.8249	1.52E+02	1.65E+02	160.5336	**148.0879**	248.8265	188.2608
	Time	8.491 s	**7.603 s**	7.975 s	10.507 s	10.306 s	11.282 s	10.517 s
F21	Mean	**2.37E+03**	2.38E+03	2.45E+03	2.61E+03	2.70E+03	2.66E+03	2.50E+03
	Std	**15.1045**	2.24E+01	4.26E+01	22.3865	98.8543	48.0492	49.3209
	Time	11.155 s	12.957 s	**8.774 s**	11.492 s	11.917 s	13.729 s	11.259 s
F22	Mean	**2.30E+03**	4.55E+03	5.70E+03	9.09E+03	6.91E+03	8.46E+03	6.09E+03
	Std	**3.4659**	2.24E+03	2.15E+03	2.44E+03	1.29E+03	1.25E+03	1.56E+03
	Time	**9.910 s**	10.985 s	12.559 s	12.049 s	13.538 s	14.414 s	12.210 s
F23	Mean	**2.72E+03**	2.73E+03	2.80E+03	3.09E+03	3.60E+03	3.17E+03	2.83E+03
	Std	**20.9042**	21.8163	3.98E+01	49.963	1.29E+02	110.3316	40.5196
	Time	10.563 s	**9.215 s**	10.309 s	12.646 s	14.247 s	13.041 s	12.723 s
F24	Mean	**2.89E+03**	2.90E+03	2.95E+03	3.25E+03	4.16E+03	3.26E+03	2.99E+03
	Std	**16.6963**	27.0045	3.53E+01	31.1493	251.4123	99.9632	37.488
	Time	10.283 s	**9.549 s**	11.158 s	13.208 s	15.920 s	14.569 s	13.848 s
F25	Mean	2.92E+03	**2.91E+03**	2.95E+03	3.67E+03	6.06E+03	3.21E+03	3.25E+03
	Std	21.9976	**1.48E+01**	3.39E+01	339.7615	5.53E+02	94.6752	321.6041
	Time	10.871 s	**10.618 s**	11.939 s	12.313 s	13.811 s	14.465 s	13.016 s
F26	Mean	**3.90E+03**	4.28E+03	4.96E+03	7.83E+03	1.18E+04	8.55E+03	5.71E+03
	Std	1.17E+03	5.48E+02	1.35E+03	**345.4336**	1.02E+03	937.3668	424.4831
	Time	12.704 s	**11.651 s**	11.872 s	14.155 s	16.730 s	15.049 s	14.176 s
F27	Mean	3.23E+03	**3.22E+03**	3.27E+03	3.55E+03	4.37E+03	3.47E+03	3.26E+03
	Std	13.421	**1.06E+01**	3.41E+01	82.9742	3.31E+02	159.0451	21.3432
	Time	15.544 s	15.010 s	**13.255 s**	14.438 s	19.309 s	16.509 s	15.161 s
F28	Mean	3.28E+03	**3.26E+03**	3.30E+03	4.37E+03	8.23E+03	3.87E+03	4.35E+03
	Std	**20.5387**	2.76E+01	3.59E+01	306.6997	515.4607	219.6228	985.7202
	Time	**9.804 s**	10.623 s	11.369 s	13.707 s	16.001 s	10.595 s	13.946 s
F29	Mean	3.80E+03	**3.77E+03**	4.38E+03	5.19E+03	1.23E+04	5.36E+03	4.26E+03
	Std	**162.1826**	1.81E+02	2.92E+02	265.2462	5.89E+03	478.9612	306.377
	Time	12.192 s	10.824 s	**9.874 s**	12.124 s	13.613 s	10.799 s	12.349 s
F30	Mean	**1.58E+04**	1.90E+04	1.11E+07	2.08E+08	1.88E+09	7.26E+07	4.76E+05
	Std	**8.34E+03**	1.25E+04	1.07E+07	8.79E+07	1.32E+09	6.00E+07	8.50E+05
	Time	18.140 s	**16.821 s**	17.467 s	19.957 s	28.995 s	27.222 s	20.526 s
Average rank		**1.38**	1.69	3.14	5.48	6.79	5.45	4.07
Rank		**1**	2	3	6	7	5	4

It is clear from Table 4 that the algorithm IEO is superior to other methods. Specifically, the average value of fitness obtained by IEO reaches the minimum when optimizing 18 functions (F3-F5, F10-F12, F14-F19, F21-F24, F26 and F30). In addition, the original EO algorithm achieves the lowest average fitness values for the 10 functions (F6-F9, F13, F20, F25, F27-29). From the experimental data of CEC2017, it can be seen that IEO is more efficient than the original EO algorithm, especially for hybrid functions and composition functions, IEO algorithm has superior performance, which indicates that IEO can avoid premature convergence to effectively solve the function optimization problem. Moreover, it can be seen from the data in the table that the running time of IEO and EO is the same order of magnitude, which indicates that IEO algorithm has excellent performance in optimizing complex functions. For the complex and challenging function set CEC2017, IEO can avoid falling into local optimal values when optimizing complex functions, so the convergence accuracy and running time of IEO are significantly improved.

**Fig 9:**
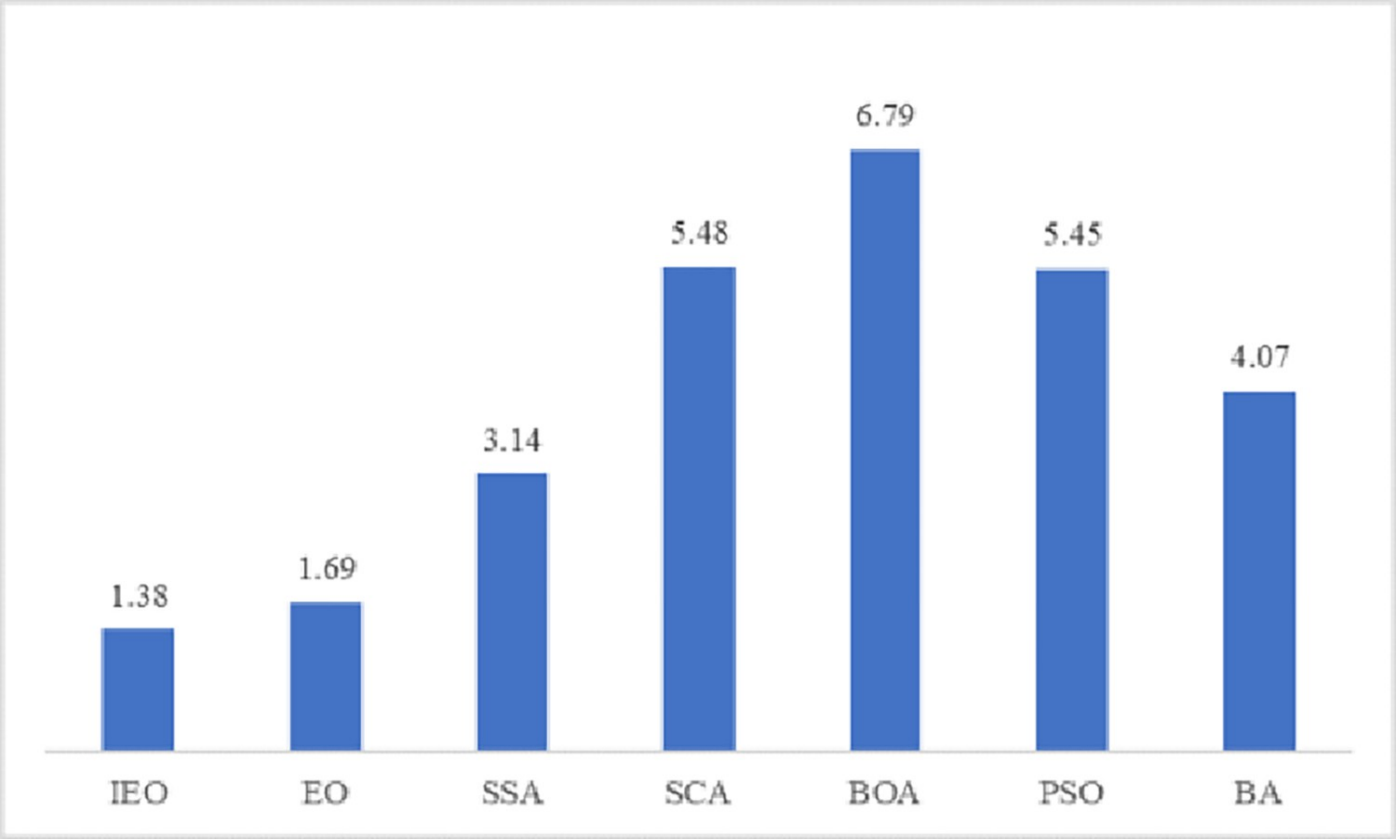
Mean rank of Friedman test on CEC2017 functions.

As shown in Fig 9, for the Friedman statistical test, the value of IEO’s mean rank is 1.38, which is much better than the value of the original EO algorithm. According to the Friedman test results in Fig 9, IEO ranks best, followed by EO, SSA, BA, PSO, SCA, and BOA, which indicates that the improved Equilibrium Optimizer has strong stability and feasibility. These results also indicate that IEO has higher convergence accuracy when optimizing the CEC2017 test suite. The Tent chaotic map and LOBL strategy has a strong global search ability, and the dynamic parameter strategy has a strong local search ability, so that the improved algorithm can effectively balance the global search ability and the local search ability to find the optimal solution.

### 3.3. IEEE CEC2019 functions

In this section, the more challenging test suite IEEE CEC2019 [33] is selected to evaluate the algorithms. These test functions are minimal and scalable. As shown in Table 5, the functions F1, F2 and F3 are 9-dimensional, 16-dimensional and 18-dimensional problems respectively, and they have different value ranges. Moreover, the functions F4-F10 are all 10-dimensional problems and have the same search scope [–100,100]. In addition, the functions F4-F10 have different rotation matrices. In the experiment, IEO is compared with Equilibrium Optimizer (EO) [18], salp swarm algorithm(SSA) [25], sine and cosine algorithm (SCA) [23], butterfly optimization algorithm (BOA) [26], the particle swarm optimization (PSO) [14] and bat algorithm (BA) [27]. For all the algorithms, the population number of each algorithm is set to 30, the maximum iteration number is set to 500, and all the algorithms are independently run on each function for 30 times, the average value (mean), standard deviation (std) and running time (time) of the fitness value are recorded. The results are shown in Table 6.

**Table 5:** Description of CEC2019 benchmark functions.

No.	Function	F_i_*=F_i_(x*)	D	Search range
1	Storn’s Chebyshev Polynomial Fitting Problem	1	9	[–8192,8192]
2	Inverse Hilbert Matrix Problem	1	16	[–16384,16384]
3	Lennard-Jones Minimum Energy Cluster	1	18	[–4,4]
4	Rastrigin’s Function	1	10	[–100,100]
5	Griewangk’s Function	1	10	[–100,100]
6	Weierstrass Function	1	10	[–100,100]
7	Modified Schwefel’s Function	1	10	[–100,100]
8	Expanded Schaffer’s F6 Function	1	10	[–100,100]
9	Happy Cat Function	1	10	[–100,100]
10	Ackley Function	1	10	[–100,100]

**Table 6:** The comparison results of different algorithms on CEC2019 functions.

Function	Result	IEO	EO	SSA	SCA	BOA	PSO	BA
F1	Mean	**1**	1	2.22E+06	6.94E+06	1	1.22E+07	1.34E+07
	Std	**0**	0	2.12E+06	7.83E+06	0	1.27E+07	1.82E+07
	Time	8.247 s	8.075 s	**4.299 s**	4.771 s	6.127 s	4.625 s	4.543 s
F2	Mean	**4.4745**	4.53	1.62E+03	3.96E+03	4.991	7.59E+03	1.09E+03
	Std	0.3504	0.3657	993.4936	1.68E+03	**0.0337**	3.51E+03	1.17E+03
	Time	6.548 s	6.222 s	4.966 s	5.058 s	**4.818 s**	4.976 s	4.880 s
F3	Mean	**1.5117**	1.5596	4.2898	9.2874	6.2543	4.8255	7.1059
	Std	**0.3153**	0.4958	1.7503	1.3239	0.8528	1.8222	2.0162
	Time	**4.056 s**	4.193 s	4.327 s	4.929 s	4.756 s	4.732 s	5.400 s
F4	Mean	**14.8968**	15.4703	28.7514	47.5372	85.4029	54.4605	29.7908
	Std	**6.1189**	6.4024	12.1888	8.8151	14.2692	18.5242	11.6568
	Time	3.980 s	3.826 s	3.900 s	3.902 s	5.016 s	**3.789 s**	4.354 s
F5	Mean	**1.0487**	1.055	1.1848	10.1705	108.6633	2.4557	2.617
	Std	**0.0361**	0.0422	0.1081	3.2055	23.6808	0.6545	4.5385
	Time	**3.837 s**	4.122 s	3.951 s	4.036 s	5.204 s	3.890 s	4.281 s
F6	Mean	**1.6811**	1.8179	4.8786	7.8184	9.0551	9.0917	4.8567
	Std	**0.7109**	0.7468	1.9377	1.0821	0.9323	1.9221	1.7535
	Time	20.363 s	20.349 s	**18.516 s**	18.781 s	35.146 s	19.299 s	19.221 s
F7	Mean	**753.474**	858.0009	1.10E+03	1.62E+03	1.94E+03	1.39E+03	995.6335
	Std	258.7518	312.3589	327.5567	234.8356	**185.5797**	375.4894	346.0445
	Time	7.232 s	7.289 s	4.404 s	**4.120 s**	5.740 s	5.133 s	4.354 s
F8	Mean	**3.7105**	3.7587	4.282	4.5317	4.8326	4.6369	4.4854
	Std	0.445	0.4918	0.4283	0.2465	**0.196**	0.3081	0.3704
	Time	6.713 s	6.935 s	4.000 s	**3.970 s**	5.447 s	4.807 s	4.199 s
F9	Mean	**1.1791**	1.187	1.3712	1.6572	4.2831	1.4193	1.3426
	Std	**0.0551**	0.0687	0.1629	0.1551	0.4278	0.1465	0.2017
	Time	4.601 s	4.523 s	3.832 s	**3.757 s**	4.856 s	4.627 s	4.032 s
F10	Mean	**17.5907**	18.0956	20.3658	21.496	21.5022	21.2662	21.1728
	Std	7.6927	7.43	3.6585	0.0898	**0.086**	0.1105	0.143
	Time	**4.234** s	4.453 s	4.520 s	4.649 s	5.259 s	4.843 s	4.473 s
Average rank		**1.10**	2.00	3.60	5.70	5.80	5.50	4.30
Rank		**1**	2	3	6	7	5	4

As shown in Table 6, it is obvious that the IEO obtains the minimum average value of the fitness values of the ten functions F1-F10. In particular, compared with the original EO algorithm, the optimization accuracy of the improved IEO algorithm for the CEC2019 functions has been improved to varying degrees. In other words, IEO is significantly better than other algorithms in optimizing the CEC2019 function. Moreover, the running time of IEO and EO is the same order of magnitude, and there is almost no difference in the running time of the two.

**Fig 10:**
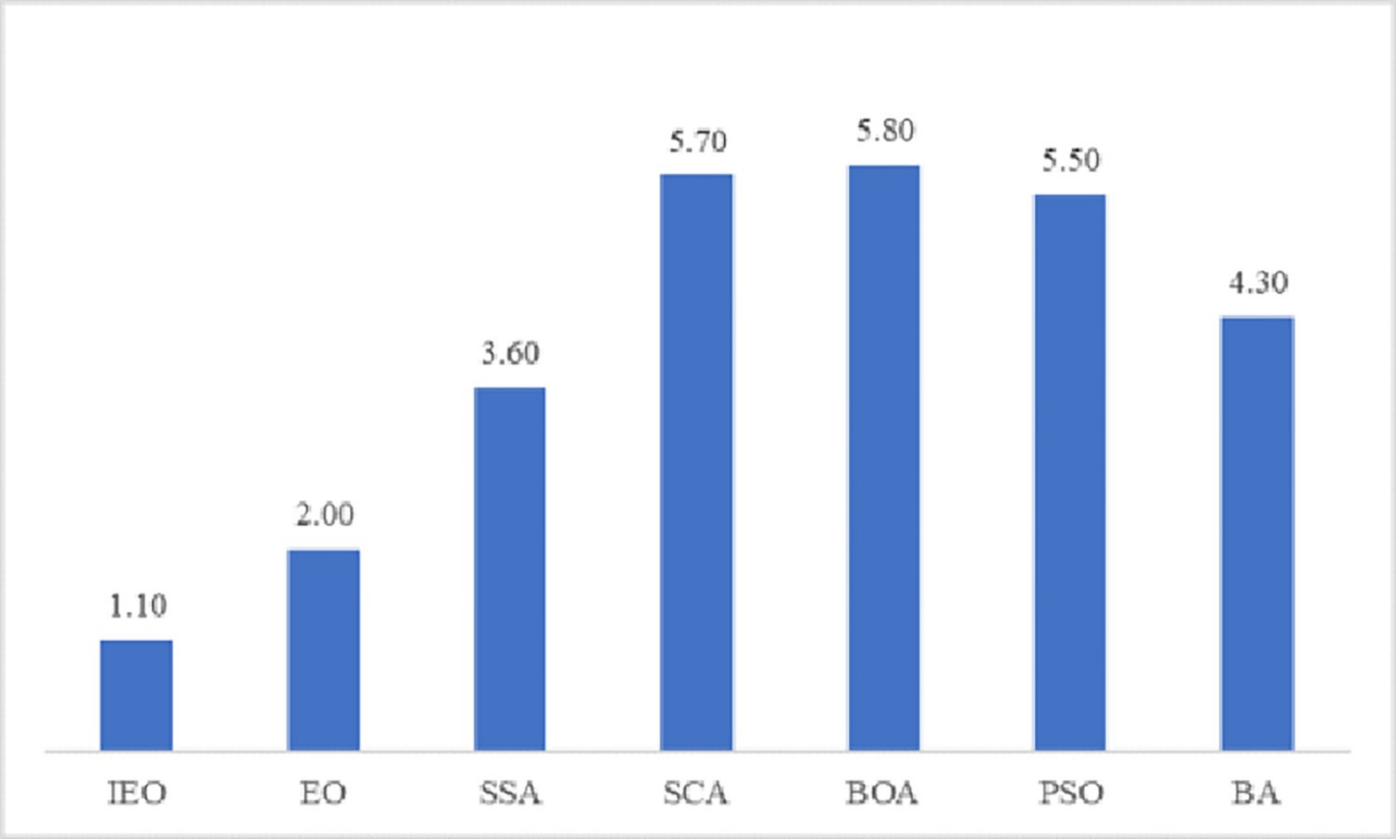
Mean rank of Friedman test on CEC2019 functions.

In addition, IEO has a mean rank of 1.1 in the Friedman statistical test, which is smaller than the other six comparison algorithms. The Fig 10 plots the average rank of each algorithm obtained in Friedman test. It can be clearly seen that IEO ranks first, while EO, SSA, BA, PSO, SCA and BOA rank second to seventh. Overall, it is clear from function optimization and Friedman test that IEO performs well with CEC2019. For the function suite CEC2019 with different rotation matrices, IEO improves the convergence accuracy when optimizing these functions. This further shows that IEO has excellent performance in complex functions.

In conclusion, the experimental result can prove adding three strategies into the Equilibrium Optimizer, the robustness and solution accuracy of the algorithm is improved, and the performance of EO has the significant improvement. Comparison with other algorithm, the IEO has better accuracy and speed to solve numerical optimization problems.

### 3.4. Analysis of convergence curve

**Fig 11:**
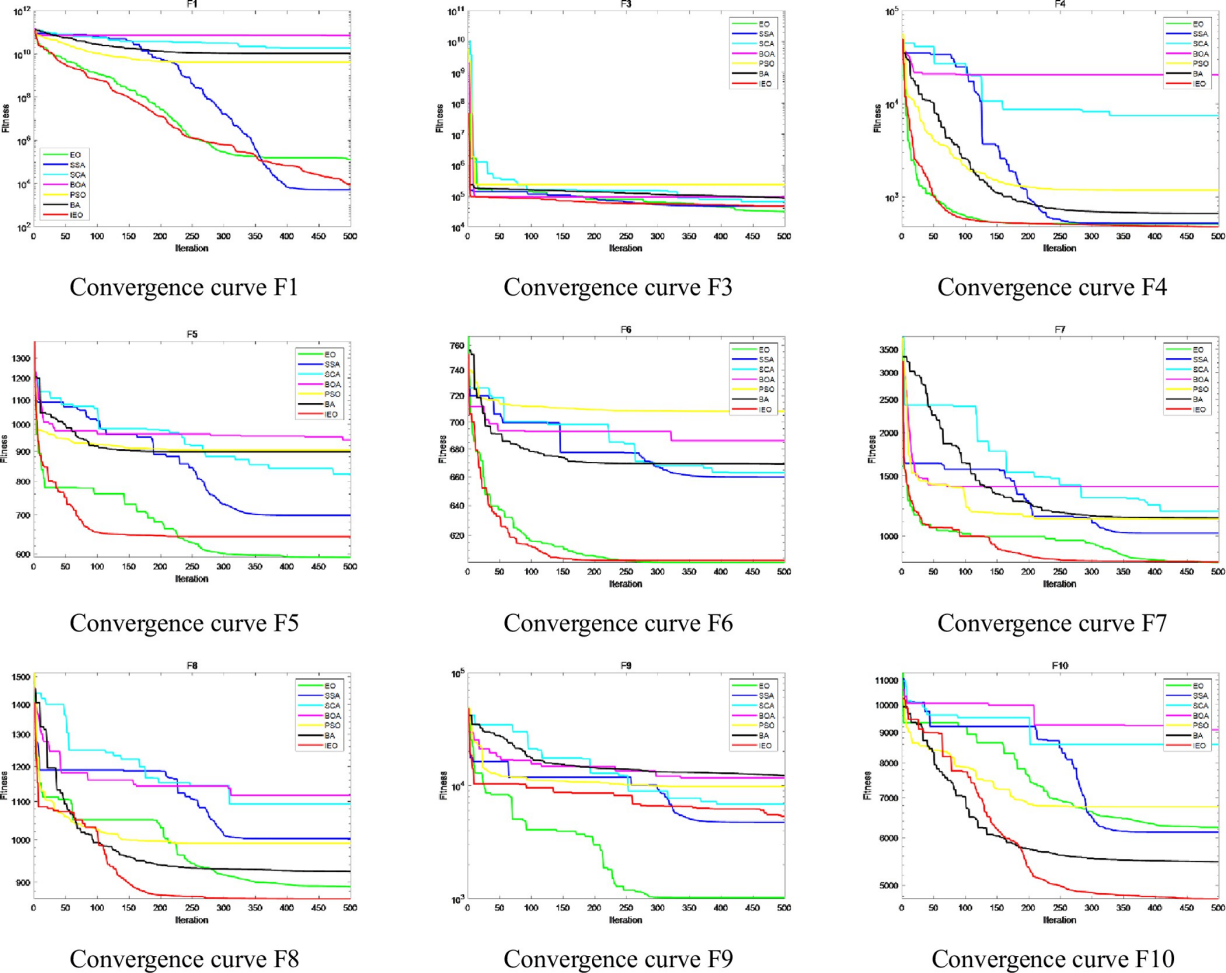
Convergence curve of CEC2017 functions: Shifted and rotated functions (F1, F3-F10).

**Fig 12:**
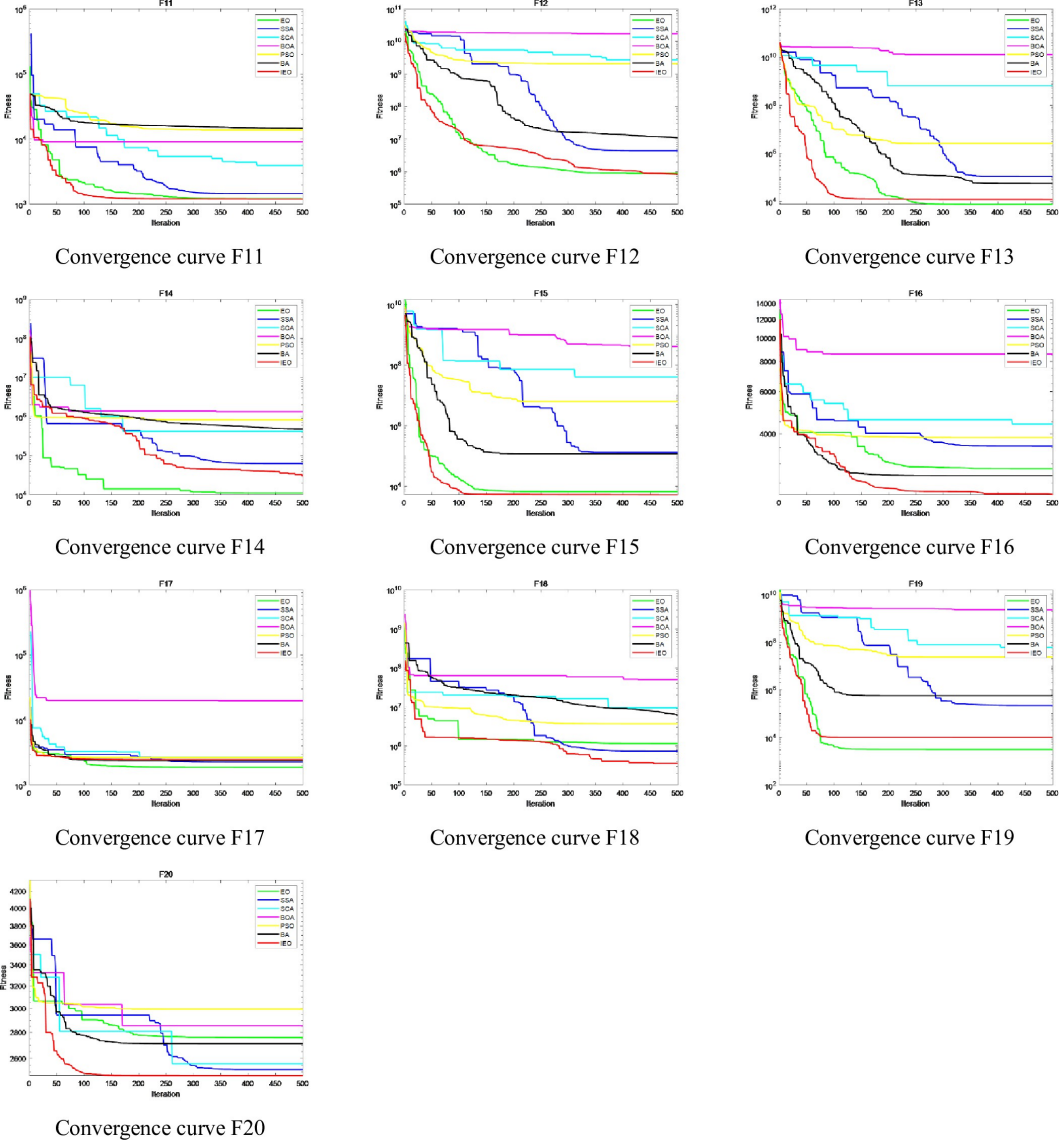
Convergence curve of CEC2017 functions: Hybrid functions (F11-F20).

**Fig 13:**
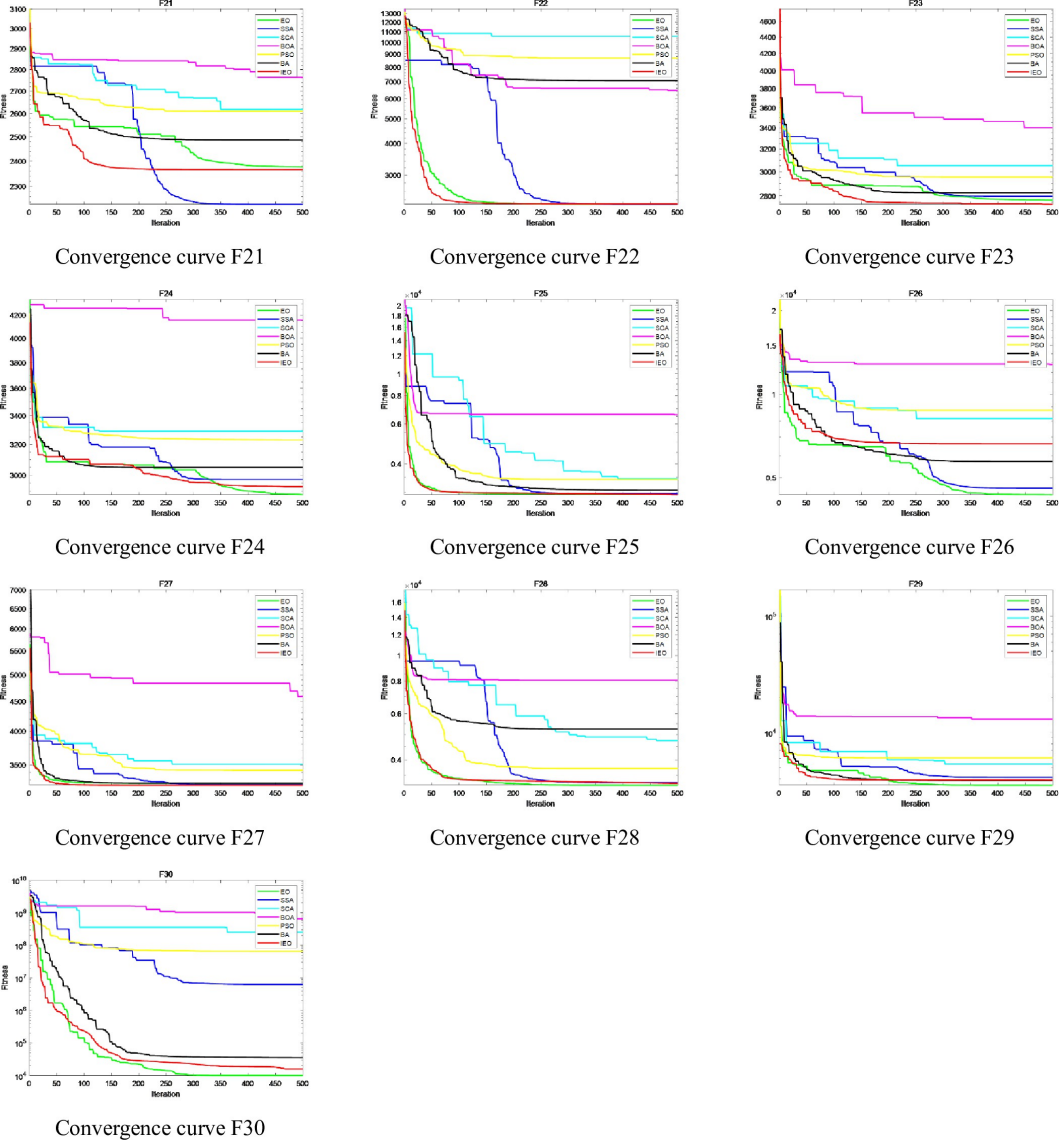
Convergence curve of CEC2017 functions: Composition functions (F21-F30).

The convergence speed and accuracy of each algorithm can be seen in detail from the convergence curve. The CEC2017 test suite contains functions of different complexity levels, which best represent the optimization performance of the algorithm. Therefore, this section analyzes the convergence status of each algorithm by drawing the convergence curve of the algorithm when optimizing the CEC2017 test suite. CEC2017 can be divided into three types of functions: shifted and rotated functions (F1, F3-F10), hybrid functions(F11-F20) and composition functions(F21-F30). According to the above three different types of functions, so three figures are drawn: Fig 11 describes the convergence curve of the shifted and rotated functions (F1, F3-F10), Fig 12 shows the convergence curve of hybrid functions (F11-F20), and Fig 13 shows the convergence curve of the composition functions (F21-F30). The Figs 11–13 describe the convergence results of IEO, EO, SSA, SCA, BOA, PSO and BA algorithms when the dimension D=30. In the figure, the horizontal axis represents the maximum iteration times of the algorithm 500 times, and the vertical axis represents the log mode of fitness value obtained in the optimization process of the algorithm.

It is clear from the figure that IEO has improved significantly for most functions. For other algorithms, EO has the best convergence, followed by SSA, PSO, BA, SCA and BOA.

For shifted and rotated functions (F1, F3-F10), the convergence speed and accuracy of IEO algorithm are obviously better than other algorithms except for functions F1, F5 and F9. It can be seen from the convergence curve that IEO converges faster and reaches the minimum fitness value at the early stage of iteration. In Fig 11, SCA has a slightly special convergence curve for some functions. It can be clearly seen from the figure that for shifted and rotated functions F1 and F3-F10, SCA has a slow convergence speed, and compared with other algorithms, SCA has a slow iteration speed and is easy to fall into the local optimal solution, which leads to a large change in the convergence curve. SCA algorithm is easy to fall into local optimization and cannot get the optimal value in a short iteration period, which leads to poor stability of the convergence curve and makes the convergence curve of SCA show significant changes. This shows that SCA is not effective in solving complex functions.

For the hybrid functions (F11-F20), although the convergence curve of IEO algorithm differs little from that of other algorithms, the convergence curve of IEO algorithm is relatively smooth and can form fast convergence within 100 iterations, showing good performance in terms of convergence speed. The improved algorithm has a strong global search ability for finding the better optimal value.

For the composition functions (F21-F30), the convergence curve of IEO algorithm is better than other algorithms except for the functions F21 and F26. In addition, the step size of IEO algorithm is obviously smaller than that of other algorithms, and it can always converge to the optimal solution at the beginning of iteration, while other algorithms tend to fall into the local optimal solution, resulting in slower convergence speed and lower convergence accuracy.

In general, convergence curve of the seven algorithms shows that IEO algorithm has significantly better convergence curve in optimizing CEC2017 function. When dealing with hybrid and composition functions, IEO has faster convergence speed and higher convergence accuracy. Especially, from some functions, the IEO can obtain the optimal value in 100 iterations. The convergence curve of CEC2017 also verifies the results in Table 4 again.

### 3.5. Stability analysis

**Fig 14:**
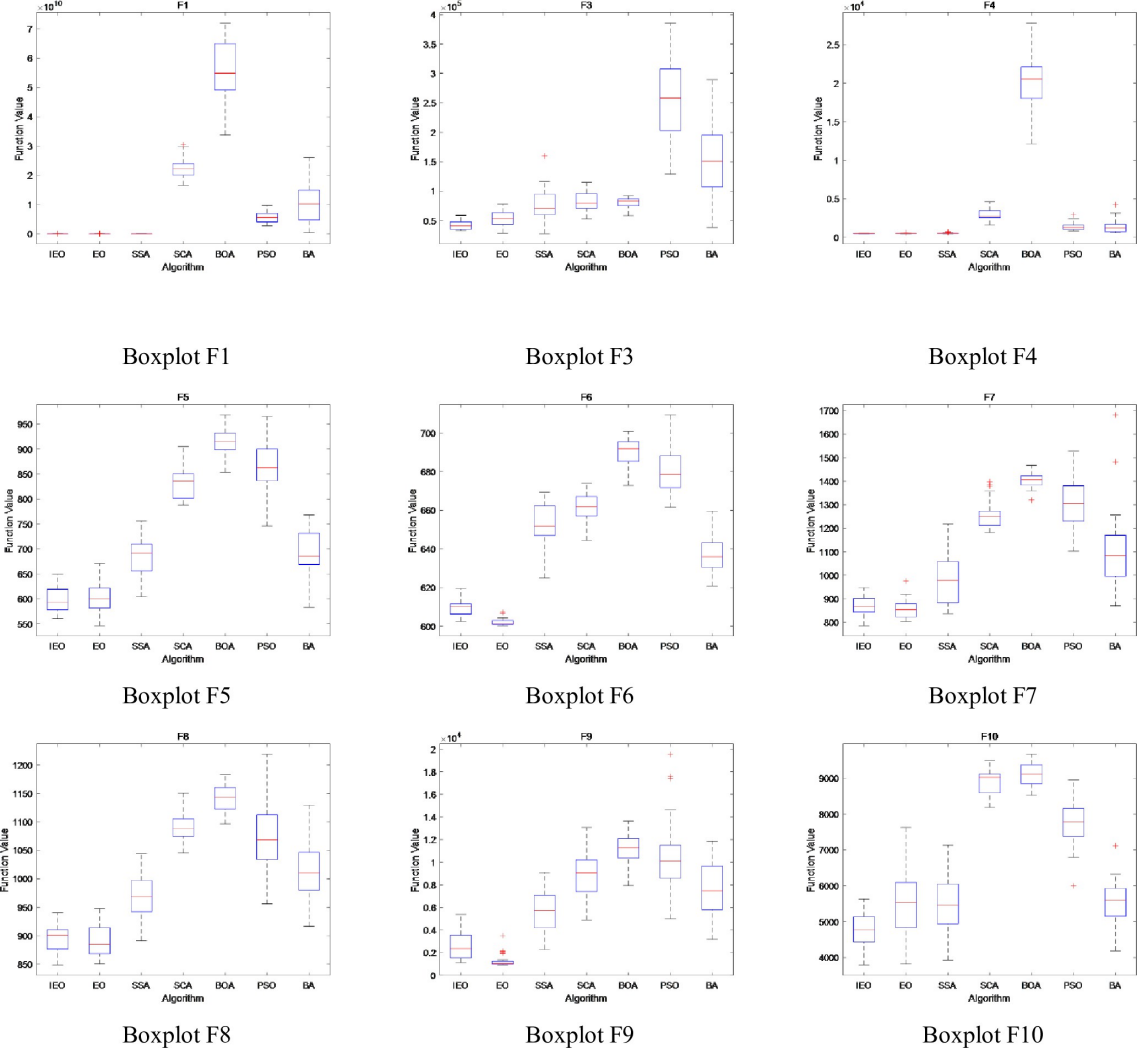
The boxplot of CEC2017 functions: Shifted and rotated functions (F1, F3-F10).

**Fig 15:**
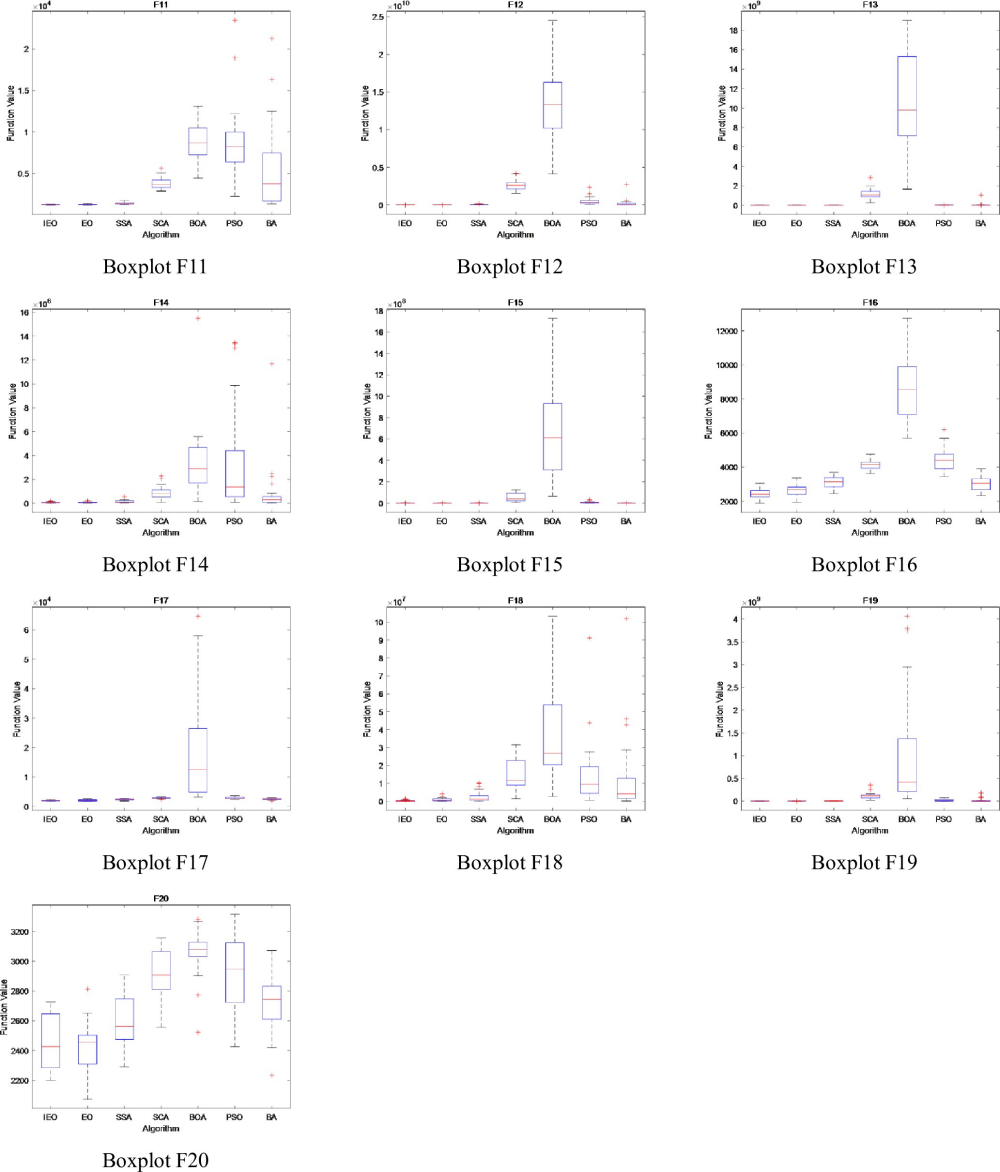
The boxplot of CEC2017 functions: Hybrid functions(F11-F20).

**Fig 16:**
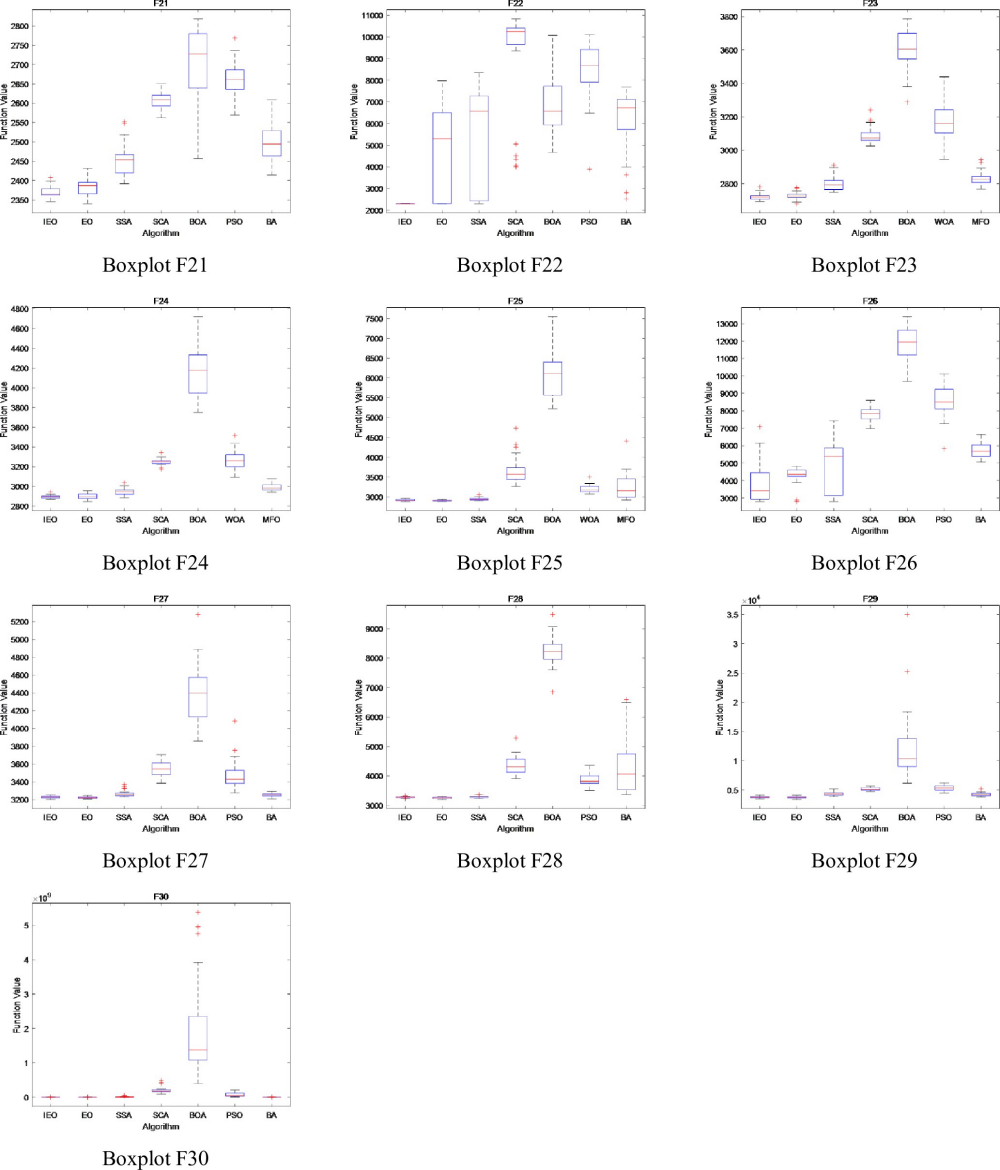
The boxplot of CEC2017 functions: Composition functions(F21-F30).

In this section, the boxplot is used to show the data distribution of CEC2017 test suite running for 30 times independently. It describes the stability and optimization performance of experimental data by using five statistics such as the maximum value, minimum value, upper quartile, lower quartile and median in the data [34]. In addition, boxplot can not only reflect the fluctuation degree of data through the height of box, but also show the stability of data through the number of outliers. The Figs 14–16 show the boxplot when dimension is set to 30, population number is 30, and maximum number of iterations is 500. Among them, Fig 14 describes the boxplot of the shifted and rotated functions (F1, F3-F10), Fig 15 shows the boxplot of hybrid functions(F11-F20), and Fig 16 shows the boxplot of the composition functions(F21-F30). In the figure, the horizontal axis represents each comparison algorithm, and the vertical axis represents the range of fitness values obtained by the optimization function.

For unimodal and multimodal functions (F1, F3-F10), except for functions F6, F7 and F9, the differences between the maximum, minimum and median of five indexes of IEO algorithm in the boxplot are the smallest, and there are no outliers, which indicates that IEO algorithm has strong stability in the process of optimizing unimodal and multimodal functions.

By observing the boxplot of hybrid functions (F11-F20), it can be seen that the data difference of IEO algorithm is small, and the number of outliers is also small, which indicates that IEO has strong stability when processing the hybrid functions. For function F20, although the difference between BOA’s maximum value and minimum value is the smallest, BOA produces a large number of outliers in the optimization process, while IEO does not produce outliers when processing function F20, and its median value is the smallest among seven algorithms.

Compared with other algorithms, the IEO algorithm is the most stable when optimizing the composition functions (F21-F30). Specifically, in the process of running IEO algorithm for 30 times independently, the data difference is the smallest, and the median value is also the smallest among the seven algorithms, which indicates that IEO algorithm has superior optimization performance.

Overall, the boxplot shows that IEO has better performance. In the initial phase of IEO algorithm, Tent chaotic mapping is introduced to improve the quality of initial solution, and dynamic control parameter strategy is introduced to maintain the balance between exploration and exploitation phase in the iterative process, so that the particles can reach balance state more effectively. In addition, the LOBL strategy is used to calculate the opposite solution of candidate solution for each iteration, which improves the population diversity. Therefore, compared with the original EO algorithm, the stability and optimization performance of the improved IEO algorithm are greatly improved.

### 3.6. Wilcoxon rank sum test analysis

In order to further analyze the significance of experimental data from a statistical perspective, Wilcoxon rank sum test is conducted at the significance level of 5% in this section [35]. Through statistical analysis of each two groups of sample data, the p-value and h-value obtained are used as indicators to evaluate whether each algorithm has statistical significance. In the experiment, if p-value<0.05 and h =1 are obtained, it means that the data of the two groups are statistically significant different [36]. For three groups of functions with different characteristics (23 classical functions, IEEE CEC2017 and IEEE CEC2019), the comparison results of IEO and other six algorithms are shown in Tables 7–9 respectively.

**Table 7:** The results of Wilcoxon rank sum test on 23 benchmark functions with D=30.

Function		IEO vs.					
		EO	SSA	SCA	BOA	PSO	BA
F1	p-value	1.2118e-12	1.2118e-12	1.2118e-12	1.2078e-12	1.2118e-12	1.2118e-12
	h	1	1	1	1	1	1
F2	p-value	3.0199e-11	3.0199e-11	3.0199e-11	3.0161e-11	3.0199e-11	3.0199e-11
	h	1	1	1	1	1	1
F3	p-value	1.4043e-12	1.4050e-12	1.4057e-12	1.3947e-12	1.4057e-12	1.4057e-12
	h	1	1	1	1	1	1
F4	p-value	3.0161e-11	3.0199e-11	3.0199e-11	3.0180e-11	3.0199e-11	3.0199e-11
	h	1	1	1	1	1	1
F5	p-value	0.1494	5.0723e-10	3.0199e-11	3.0199e-11	3.0199e-11	3.0199e-11
	h	0	1	1	1	1	1
F6	p-value	0.0011	3.6874e-11	3.0180e-11	3.0180e-11	3.0180e-11	3.0180e-11
	h	1	1	1	1	1	1
F7	p-value	1.2118e-12	1.2118e-12	1.2118e-12	1.1980e-12	1.2118e-12	1.2078e-12
	h	1	1	1	1	1	1
F8	p-value	0.1958	1.2023e-08	3.0199e-11	3.0199e-11	0.0292	0.0138
	h	0	1	1	1	1	1
F9	p-value	NaN	1.2039e-12	1.2118e-12	6.2385e-10	0.1608	1.2118e-12
	h	0	1	1	1	0	1
F10	p-value	8.4109e-12	1.3324e-11	1.3369e-11	1.3360e-11	6.3428e-05	1.3369e-11
	h	1	1	1	1	1	1
F11	p-value	0.3337	1.2118e-12	1.2118e-12	1.2118e-12	0.3337	1.2118e-12
	h	0	1	1	1	0	1
F12	p-value	4.1804e-09	3.0180e-11	3.0180e-11	3.0180e-11	3.0180e-11	3.0180e-11
	h	1	1	1	1	1	1
F13	p-value	2.1880e-04	1.4727e-07	3.0180e-11	8.9827e-11	0.0103	3.0180e-11
	h	1	1	1	1	1	1
F14	p-value	0.0814	0.9434	1.1248e-09	4.0051e-08	0.0016	1.5400e-04
	h	0	0	1	1	1	1
F15	p-value	0.1103	3.9966e-10	1.3273e-10	1.1557e-07	9.2516e-09	2.3472e-10
	h	0	1	1	1	1	1
F16	p-value	NAN	NAN	0.0027	1.2118e-12	NAN	NAN
	h	0	0	1	1	0	0
F17	p-value	NAN	NAN	1.2108e-12	NAN	0.0110	NAN
	h	0	0	1	NAN	1	0
F18	p-value	NAN	NAN	2.7717e-05	1.2118e-12	0.0013	NAN
	h	0	0	1	1	1	0
F19	p-value	NAN	NAN	1.2059e-12	1.3341e-08	1.6497e-11	NAN
	h	0	0	1	1	1	0
F20	p-value	0.3337	0.3337	2.4110e-12	2.5886e-06	2.3157e-10	0.3337
	h	0	0	1	1	1	0
F21	p-value	0.3321	0.3851	1.3265e-10	3.1917e-10	2.8457e-04	0.0020
	h	0	0	1	1	1	0
F22	p-value	0.3710	0.2978	5.2776e-09	2.2527e-08	7.3127e-08	0.0164
	h	0	0	1	1	1	1
F23	p-value	0.9773	0.1681	1.7566e-10	6.3054e-11	6.4169e-10	0.0019
	h	0	0	1	1	1	1

**Table 8:** The results of Wilcoxon rank sum test on CEC2017 functions with D=30.

Function		IEO vs.					
		EO	SSA	SCA	BOA	PSO	BA
F1	p-value	0.0163	1.5292e-05	3.0199e-11	3.0199e-11	3.0199e-11	3.0199e-11
	h	1	1	1	1	1	1
F3	p-value	9.0307e-04	2.1947e-08	4.9752e-11	3.3384e-11	3.0199e-11	3.5708e-06
	h	1	1	1	1	1	1
F4	p-value	0.4290	0.0339	3.0199e-11	3.0199e-11	4.1825e-09	3.0199e-11
	h	0	1	1	1	1	1
F5	p-value	0.4643	1.1737e-09	3.0199e-11	3.0199e-11	0.0207	3.0199e-11
	h	0	1	1	1	1	1
F6	p-value	1.1737e-09	3.0199e-11	3.0199e-11	3.0199e-11	1.1747e-04	3.0199e-11
	h	1	1	1	1	1	1
F7	p-value	0.1537	1.2493e-05	3.0199e-11	3.0199e-11	0.0013	3.0199e-11
	h	0	1	1	1	1	1
F8	p-value	0.4204	2.9215e-09	3.0199e-11	3.0199e-11	0.0040	3.0199e-11
	h	0	1	1	1	1	1
F9	p-value	6.5277e-08	4.3106e-08	4.0772e-11	3.0199e-11	0.0049	3.0199e-11
	h	1	1	1	1	1	1
F10	p-value	0.0012	1.4067e-04	3.0199e-11	3.0199e-11	4.9980e-09	3.0199e-11
	h	1	1	1	1	1	1
F11	p-value	0.5106	2.0338e-09	3.0199e-11	3.0199e-11	2.2273e-09	3.0199e-11
	h	0	1	1	1	1	1
F12	p-value	0.1858	6.0658e-11	3.0199e-11	3.0199e-11	2.3885e-04	3.0199e-11
	h	0	1	1	1	1	1
F13	p-value	0.4553	6.0658e-11	3.0199e-11	3.0199e-11	0.0339	3.0199e-11
	h	0	1	1	1	1	1
F14	p-value	0.1669	1.4932e-04	5.4941e-11	3.6897e-11	0.0025	3.0199e-11
	h	0	1	1	1	1	1
F15	p-value	0.9470	7.3891e-11	3.0199e-11	3.0199e-11	1.9963e-05	3.0199e-11
	h	0	1	1	1	1	1
F16	p-value	0.0303	4.9980e-09	3.0199e-11	3.0199e-11	0.0033	3.0199e-11
	h	1	1	1	1	1	1
F17	p-value	0.3478	1.4298e-05	4.0772e-11	3.0199e-11	0.6735	3.0199e-11
	h	0	1	1	1	0	1
F18	p-value	4.4592e-04	2.3168e-06	3.3384e-11	3.0199e-11	4.9980e-09	3.0199e-11
	h	1	1	1	1	1	1
F19	p-value	0.2170	3.0199e-11	3.0199e-11	3.0199e-11	1.0277e-06	3.0199e-11
	h	0	1	1	1	1	1
F20	p-value	0.9352	0.0014	5.0723e-10	7.3891e-11	0.8073	3.0199e-11
	h	0	1	1	1	0	1
F21	p-value	0.0087	8.9934e-11	3.0199e-11	3.0199e-11	1.2860e-06	3.0199e-11
	h	1	1	1	1	1	1
F22	p-value	1.5846e-04	4.6856e-08	3.0199e-11	3.0199e-11	3.0199e-11	3.0199e-11
	h	1	1	1	1	1	1
F23	p-value	0.1494	3.1589e-10	3.0199e-11	3.0199e-11	3.8053e-07	3.0199e-11
	h	0	1	1	1	1	1
F24	p-value	0.5895	7.7725e-09	3.0199e-11	3.0199e-11	5.4941e-11	3.0199e-11
	h	0	1	1	1	1	1
F25	p-value	0.0428	3.9859e-04	3.0180e-11	3.0180e-11	0.0070	3.0180e-11
	h	1	1	1	1	1	1
F26	p-value	0.0451	0.0035	3.3384e-11	3.0199e-11	5.2650e-05	3.0199e-11
	h	1	1	1	1	1	1
F27	p-value	0.0421	2.3887e-08	3.0180e-11	3.0180e-11	1.0930e-10	6.3599e-07
	h	1	1	1	1	1	1
F28	p-value	5.8696e-04	0.0537	3.0123e-11	3.0123e-11	0.0046	6.0111e-08
	h	1	0	1	1	1	1
F29	p-value	0.4918	4.1997e-10	3.0199e-11	3.0199e-11	0.8187	3.0199e-11
	h	0	1	1	1	0	1
F30	p-value	0.3042	3.0199e-11	3.0199e-11	3.0199e-11	6.5277e-08	3.0199e-11
	h	0	1	1	1	1	1

**Table 9:** The results of Wilcoxon rank sum test on CEC2019 functions.

Function		IEO vs.					
		EO	SSA	SCA	BOA	PSO	BA
F1	p-value	NaN	1.2118e-12	1.2118e-12	NaN	1.2118e-12	NaN
	h	0	1	1	0	1	0
F2	p-value	0.0480	2.7687e-11	2.7687e-11	5.5233e-06	2.7687e-11	2.0815e-07
	h	1	1	1	1	1	1
F3	p-value	0.0477	1.4130e-09	2.5303e-11	2.5303e-11	5.1096e-11	4.4498e-07
	h	1	1	1	1	1	1
F4	p-value	0.8999	4.0449e-06	4.4739e-11	2.9991e-11	0.1512	2.9991e-11
	h	0	1	1	1	0	1
F5	p-value	0.4914	2.9887e-07	2.9935e-11	2.9935e-11	7.7209e-09	2.9935e-11
	h	0	1	1	1	1	1
F6	p-value	0.4290	2.0152e-08	3.0199e-11	3.0199e-11	0.0748	3.0199e-11
	h	0	1	1	1	0	1
F7	p-value	0.0468	3.3681e-05	4.0772e-11	3.0199e-11	0.0127	3.0199e-11
	h	1	1	1	1	1	1
F8	p-value	0.6204	2.2780e-05	4.5726e-09	4.9752e-11	0.5106	3.6897e-11
	h	0	1	1	1	0	1
F9	p-value	0.0326	3.9532e-07	3.6874e-11	3.0180e-11	0.2282	3.0180e-11
	h	1	1	1	1	0	1
F10	p-value	0.8359	1.5075e-04	9.5200e-07	9.1649e-07	0.0701	6.7556e-05
	h	0	1	1	1	0	1

As can be seen from Table 7, when Wilcoxon rank sum test is performed for function F1-F13, IEO is significantly different from EO. However, IEO’s performance is not outstanding for fixed dimensional multimodal functions F14-F23. When IEO and SCA perform statistical checks, all test functions are significantly different. Since BOA cannot optimize F17, the comparison result is expressed as NAN. Compared with this algorithm, IEO has significant differences for the other 22 functions. Compared with PSO, except F9, F11 and F16, the other 20 functions showed significant differences in statistical angle. When IEO and BA perform Wilcoxon rank sum test, the other 17 functions have significant statistical differences except the six fixed-dimension functions F16-F21.

As can be seen from the statistical test results in Table 8, in the Wilcoxon rank sum test of IEO and EO, the two algorithms have significant differences for 13 functions. When IEO performs statistical tests with SSA, SCA and BOA algorithms, all functions except F28 have significant differences. Compared with PSO algorithm, the differences of other functions except F17, F20 and F29 are statistically significant. When comparing the experimental data of IEO and BA, all functions have significant statistical differences. In general, IEO is statistically significantly different from other algorithms, which indicates that IEO algorithm has higher convergence accuracy.

As can be seen from Table 9, except for function F1, when IEO is respectively compared with SSA, SCA, BOA and BA, the other 9 functions have significant differences. Compared with the classical PSO algorithm, IEO has significant statistical differences for five functions. In the statistical tests of IEO and EO, the two algorithms converge to the theoretical optimal value in the test of function F1, resulting in the representation of p-value as NAN and h-value as 0. In addition, the two algorithms have significant differences for the other four functions. In general, when optimizing the function IEEE CEC2019, the improved algorithm IEO is statistically significant different from other algorithms.

Wilcoxon rank sum test is performed on three function test sets to verify the significance of IEO algorithm from a statistical point of view, and further shows that IEO has higher convergence accuracy.

### 4. Engineering design problems

In this section, the efficiency of IEO algorithm in solving practical application problems is tested by solving six engineering problems. The solution of engineering optimization problems is to give the optimal design scheme under the premise of satisfying multiple constraints. In the experiment, the overall size is set as 30, and the maximum number of iterations is 500. IEO is compared with various meta-heuristic algorithms, and the optimal solution of each problem is shown in bold in the table.

### 4.1. Pressure vessel design problem

**Fig 17:**
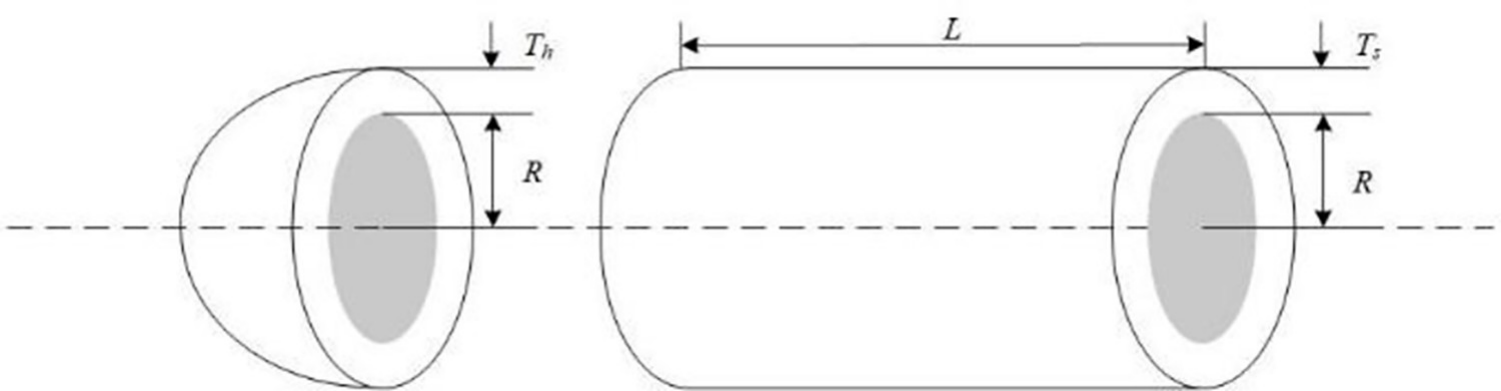
Pressure vessel design problem.

The optimization objective of pressure vessel design problem [37] is to minimize the total cost of cylindrical pressure vessels, a schematic diagram of this problem is shown in Fig 17, where four key optimization variables are involved: thickness of the head (*T*_*h*_), the thickness of the shell (*T*_*s*_), the inner radius (*R*), and the length of the cylindrical section without considering the head(*L*). The mathematical expression of this problem is as follows [38]:

Consider                $\vec{x}=[x_{1}\;x_{2}\;x_{3}\;x_{4}]=[T_{s}\;T_{h}\;R\;L]$

Minimize                $f(\vec{x})=0.6224x_{1}x_{3}x_{4}+1.7781x_{2}x_{3}^{2}+3.1661x_{1}^{2}x_{4}+19.84x_{1}^{2}x_{3}$

Subject to                $g_{1}(\vec{x})=-x_{1}+0.0193x_{3}\leq 0$

                                $g_{2}(\vec{x})=-x_{2}+0.00954x_{3}\leq 0$

                                $g_{3}(\vec{x})=-\pi x_{3}^{2}x_{4}-\frac{4}{3}\pi x_{3}^{3}+1296000\leq 0$

                                $g_{4}(\vec{x})=x_{4}-240\leq 0$

Variable range 0≤*x*_1_≤99, 0≤*x*_2_≤99, 10≤*x*_3_≤200, 10≤*x*_4_≤200

**Fig 18:**
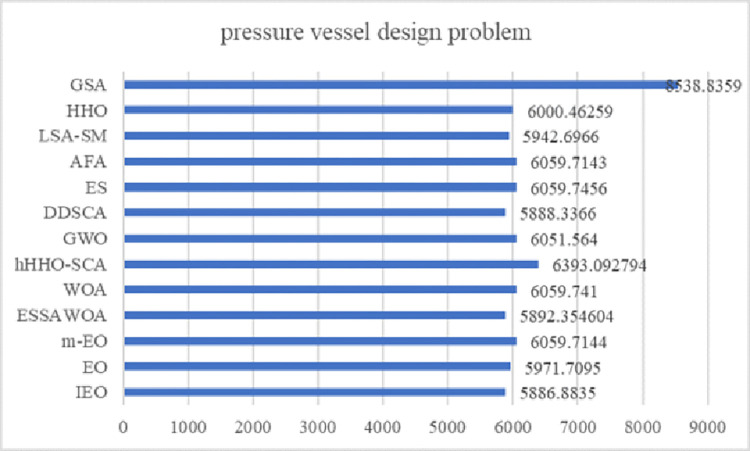
The optimal cost of pressure vessel design problem.

Four key variables of the pressure vessel problem are optimized by IEO algorithm and the optimal values are obtained, and the results are compared with the data of 12 algorithms that have solved the problem. Table 10 shows the values of the lowest costs and associated variables derived from each algorithm. To more clearly reflect the optimal cost of each algorithm, the Fig 18 is drawn.

**Table 10:** Comparison of result on pressure vessel design problem.

Algorithm	Optimal values for variables				Optimal cost
	*T*_*s*_	*T*_*h*_	*R*	*L*	
IEO	**0.7790748**	**0.3850971**	**40.36657**	**199.3474**	**5886.8835**
EO	0.8257301	0.4081611	42.78394	168.3235	5971.7095
m-EO [19]	0.8125	0.4375	42.0984	76.6366	6059.7144
ESSAWOA [39]	0.7817639	0.3864301	40.5056956	197.4631899	5892.3546036
WOA [17]	0.812500	0.437500	42.0982699	176.638998	6059.7410
hHHO-SCA [40]	0.945909	0.447138	46.8513	125.4684	6393.092794
GWO [41]	0.8125	0.4345	42.0892	176.7587	6051.564
DDSCA [42]	0.7782114	0.3855657	40.31989	200	5888.3366
ES [43]	0.8125	0.4375	42.098087	176.640518	6059.74560
AFA [44]	0.8125	0.4375	42.0984	176.6366	6059.7143
LSA-SM [45]	0.8103764	0.4005695	41.98842	178.0048	5942.6966
HHO [2]	0.8175838	0.4072927	42.09174576	176.7196352	6000.46259
GSA [9]	1.125	0.625	55.9886598	84.4542025	8538.8359

As can be seen from Table 10, IEO algorithm can obtain the minimum cost when solving the pressure vessel problem, and the values of four related parameters are relatively good, which effectively saves the engineering design cost.

### 4.2. Welded beam design problem

**Fig 19:**
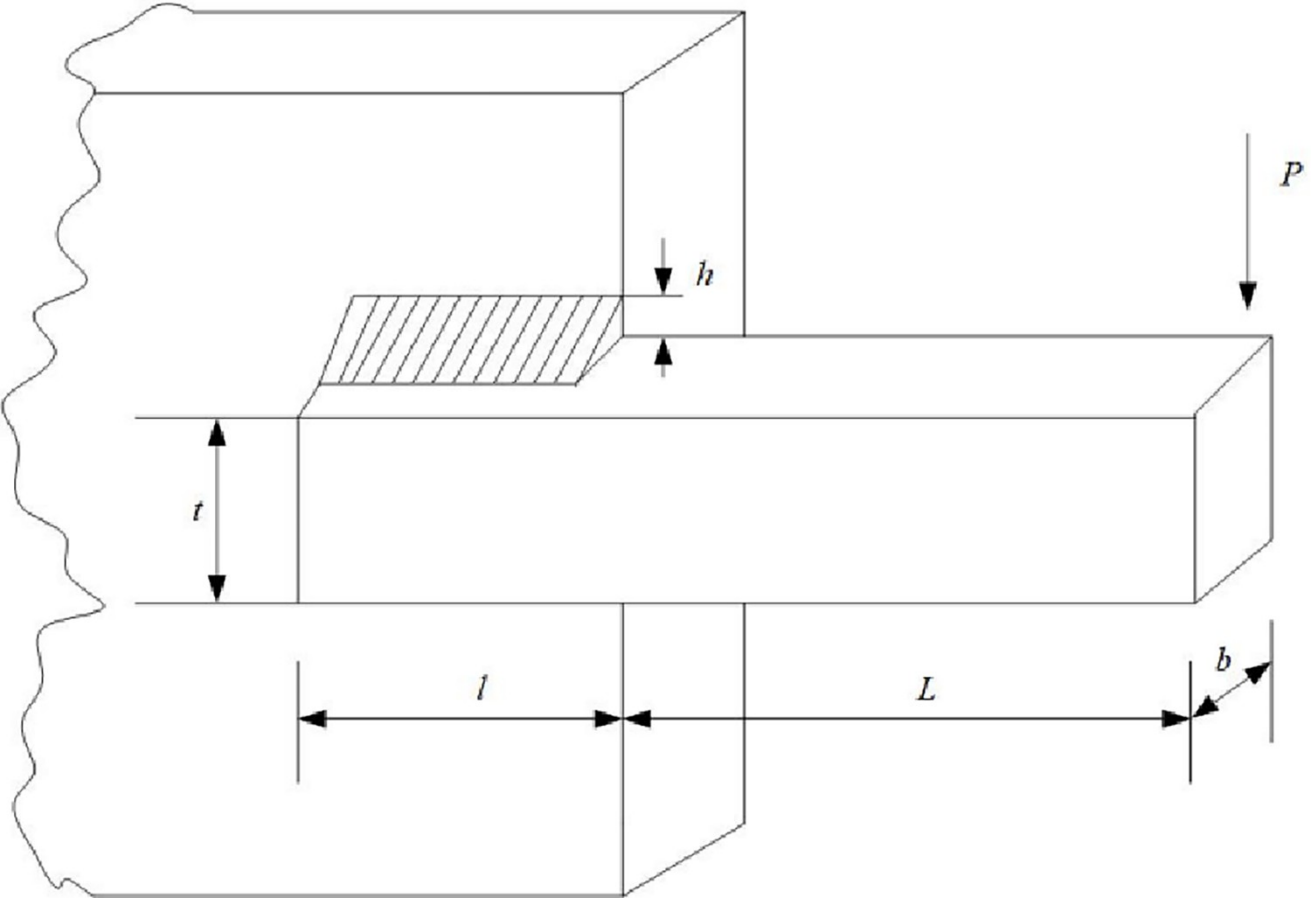
Welded beam design problem.

The objective of welded beam design problem is to minimize the manufacturing cost of welded beam design [46], as shown in Fig 19. The following constraints must be met during the optimization of welded beam problem: height (*t*), thickness of weld (*h*), length (*l*), and thickness (*b*) of the bar.

The mathematical model of this problem is expressed as follows:

Consider                $\vec{x}=[x_{1}\;x_{2}\;x_{3}\;x_{4}]=[h\;l\;t\;b]$

Minimize                $f(\vec{x})=1.10471x_{1}^{2}x_{2}+0.04811x_{3}x_{4}(14.0+x_{2})$

Subject to                $g_{1}(\vec{x})=\tau (\vec{x})-\tau_{max}\leq 0$

                                $g_{2}(\vec{x})=\sigma (\vec{x})-\sigma_{max}\leq 0$

                                $g_{3}(\vec{x})=\delta (\vec{x})-\delta_{max}\leq 0$

                                $g_{4}(\vec{x})=x_{1}-x_{4}\leq 0$

                                $g_{5}(\vec{x})=p-p_{c}(\vec{x})\leq 0$

                                $g_{6}(\vec{x})=0.125-x_{1}\leq 0$

                                $g_{7}(\vec{x})=0.10471x_{1}^{2}+0.04811x_{3}x_{4}(14.0+x_{2})-5.0\leq 0$

Variable range 0.1≤*x*_1_≤2, 0.1≤*x*_2_≤10

                                0.1≤*x*_3_≤10, 0.1≤*x*_4_≤2

where                    $\tau (\vec{x})=\sqrt{{\left(\tau^{\prime }\right)}^{2}+2\tau^{\prime }\tau^{\prime \prime }\frac{x_{2}}{2R}+{\left(\tau^{\prime \prime }\right)}^{2}},$ $\tau^{\prime }=\frac{p}{\sqrt{2}x_{1}x_{2}},\tau^{\prime \prime }=\frac{MR}{J}$

                                $M=p(L+\frac{x_{2}}{2}),$       $R=\sqrt{\frac{x_{2}^{2}}{4}+{\left(\frac{x_{1}+x_{3}}{2}\right)}^{2}}$

                                $J=2\left\{\sqrt{2}x_{1}x_{2}[\frac{x_{2}^{2}}{12}+{\left(\frac{x_{1}+x_{3}}{2}\right)}^{2}]\right\},$ $\sigma (\vec{x})=\frac{6PL}{x_{4}x_{3}^{2}},\;\delta (\vec{x})=\frac{4PL^{3}}{E{x_{3}^{3}x}_{4}}$

                                $P_{C}(\vec{x})=\frac{4.013E\sqrt{\frac{x_{3}^{2}x_{4}^{6}}{36}}}{L^{2}}\left(1-\frac{x_{3}}{2L}\sqrt{\frac{E}{4G}}\right)$

                                *p* = 6000lb, L = 14 in., *δ*_*max*_ = 0.25 *in*.

                                E = 30×10^6^psi, G = 12×10^6^psi

                                *τ*_*max*_ = 13,600*psi*, *σ*_*max*_ = 30,000*psi*

**Fig 20:**
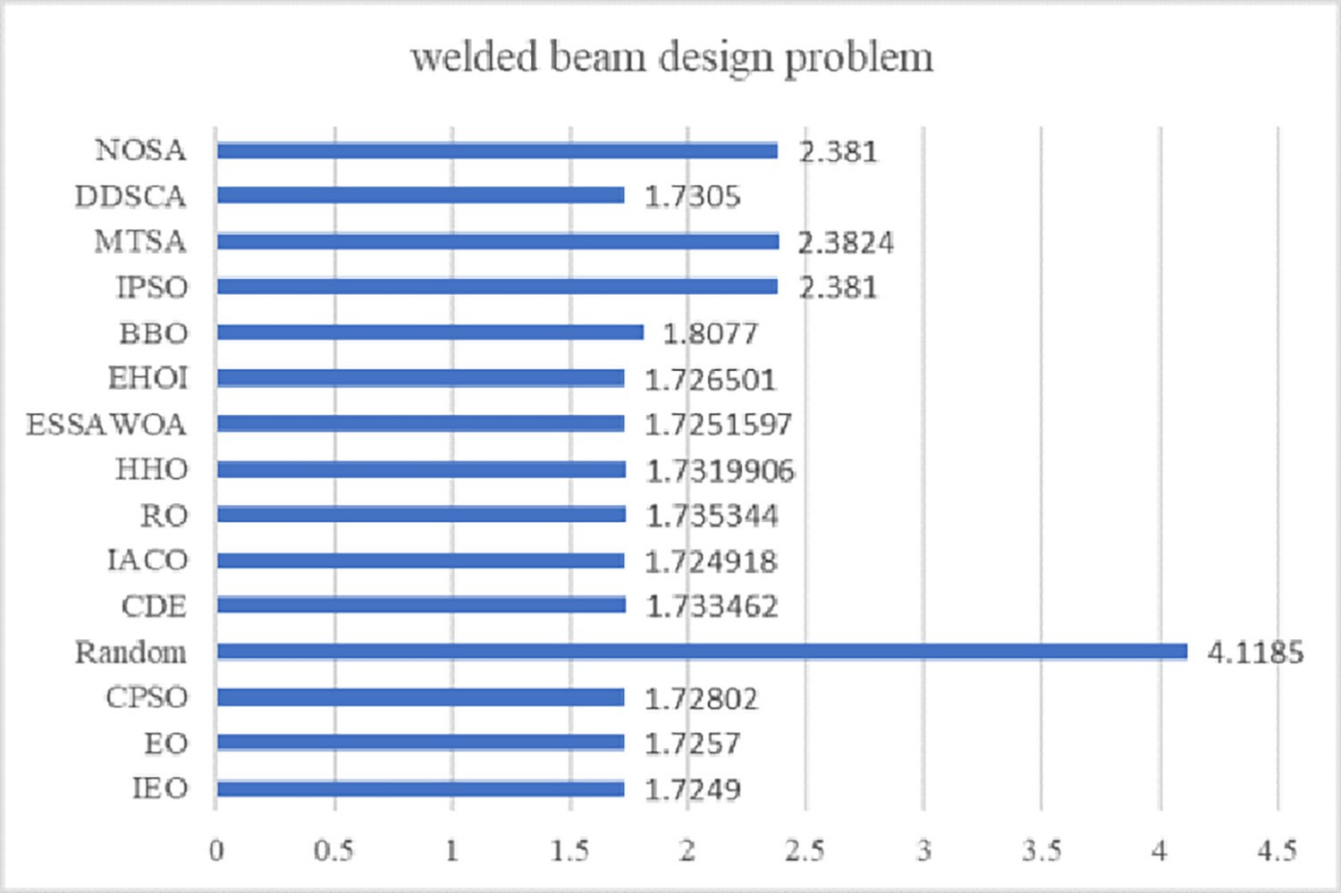
The optimal cost of welded beam l design problem.

The welded beam problem is optimized by IEO and the original EO algorithm, and the experimental results are compared with 13 algorithms in other literatures. Table 11 shows the results of different algorithms and the values of four related parameters. To more clearly reflect the optimal cost of IEO, the Fig 20 is drawn.

**Table 11:** Comparison of result on welded beam design problem.

Algorithm	Optimal values for variables				Optimal cost
	*h*	*l*	*t*	*b*	
IEO	**0.20573**	**3.4703**	**9.0372**	**0.20573**	**1.7249**
EO	0.20593	3.4681	9.0322	0.20594	1.7257
CPSO [47]	0.202369	3.544214	9.048210	0.205723	1.72802
Random [48]	0.4575	4.7313	5.0853	0.6600	4.1185
CDE [49]	0.20317	3.542998	9.033498	0.206179	1.733462
IACO [50]	0.205700	3.471131	9.036683	0.205731	1.724918
RO [51]	0.203687	3.528467	9.004263	0.207241	1.735344
HHO [2]	0.204039	3.531061	9.027463	0.206147	1.7319906
ESSAWOA [39]	0.2055051	3.4753160	9.0366562	0.2057295	1.7251597
EHOI [52]	0.205377	3.472652	9.050768	0.205659	1.726501
BBO [53]	0.2287	3.2003	8.5666	8.9985	1.8077
IPSO [54]	0.2444	6.2175	8.2915	0.2444	2.3810
MTSA [55]	0.2442	6.2231	8.2956	0.2444	2.3824
DDSCA [42]	0.20516	3.4759	9.0797	0.20552	1.7305
NOSA [56]	0.2444	6.2175	8.2915	0.2444	2.3810

It can be seen from Table 11 that the IEO algorithm has the smallest manufacturing cost for welded beam problem. In other words, when the value of four key parameters are 0.20573, 3.4703, 9.0372, 0.20573, the manufacturing cost of welded beam is 1.7249. This shows that the performance of IEO is better than other algorithms. It not only improves the optimization efficiency, but also reduces the cost of solving welded beam problem.

### 4.3. Tension/Compression spring design problem

**Fig 21:**
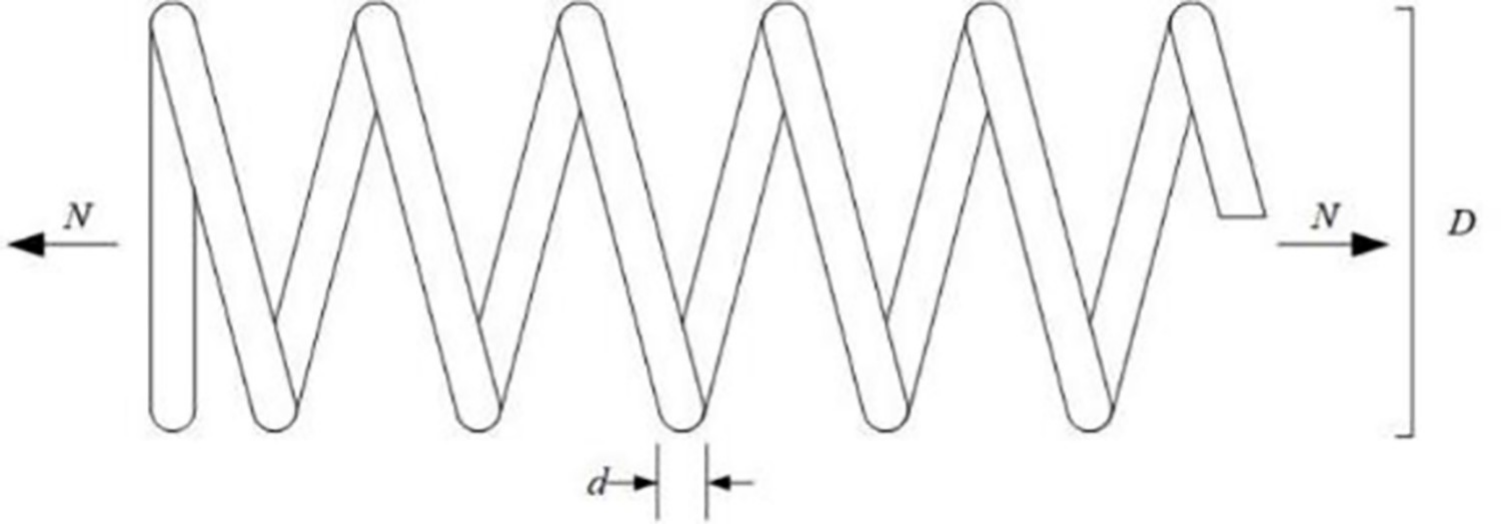
Tension/compression spring design problem.

The tension/compression spring problem is a classic structural engineering design problem [57], whose purpose is to minimize the weight of tension/compression spring. To solve the problem, three core variables are needed: wire diameter (*d*), mean coil diameter (*D*), and number of active coils (*N*) [58]. The details of the spring and the three parameters are shown in Fig 21.

**Fig 22:**
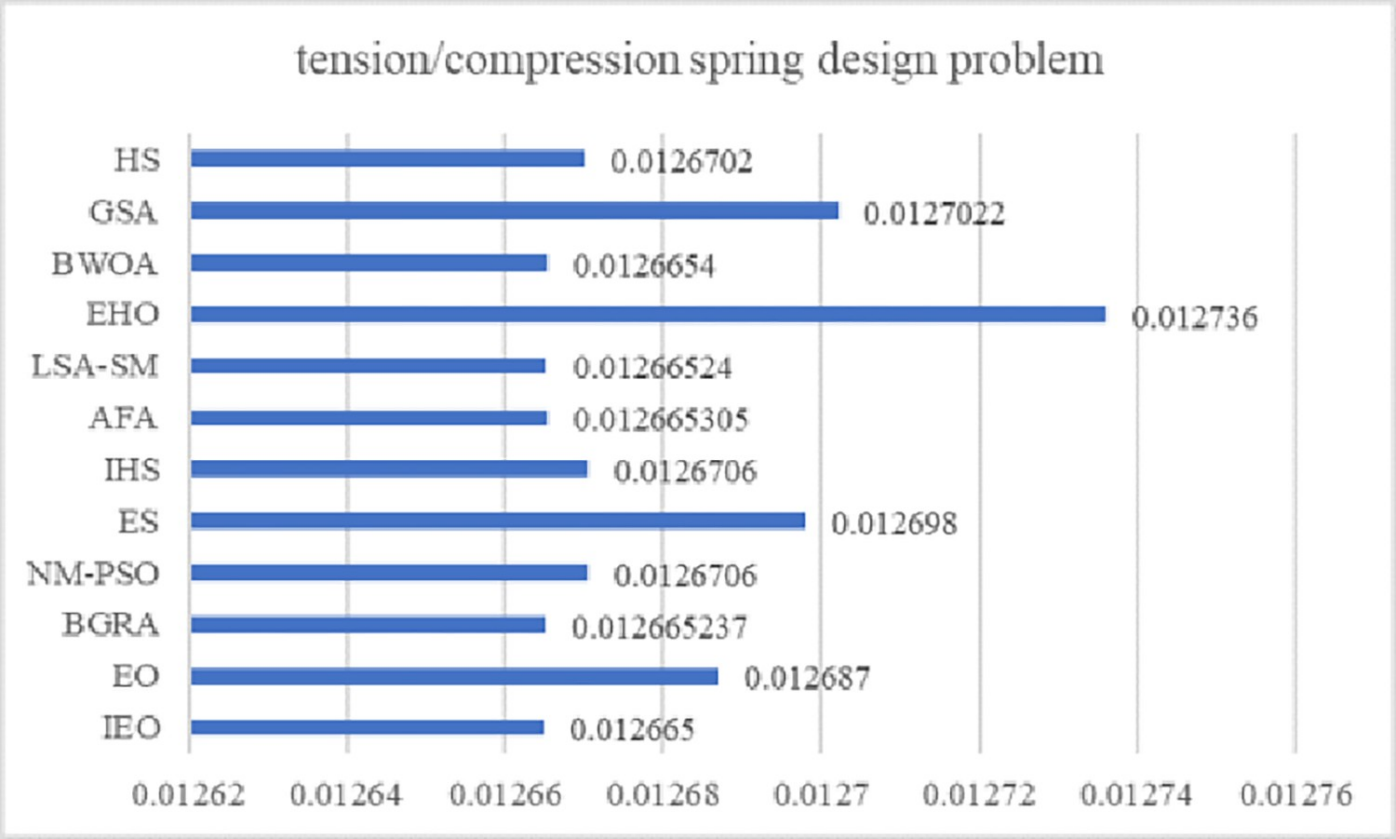
The optimal cost of tension/compression spring design problem.

The mathematical model of this problem is as follows:

Consider                $\vec{x}=[x_{1}\;x_{2}\;x_{3}]=[d\;D\;N]$,

Minimize                $f(\vec{x})=(x_{3}+2)x_{2}x_{1}^{2}$,

Subject to                $g_{1}(\vec{x})=1-\frac{x_{2}^{2}x_{3}}{71785x_{1}^{4}}\leq 0$,

                                $g_{2}(\vec{x})=\frac{4x_{2}^{2}-x_{1}x_{2}}{12566(x_{2}x_{1}^{3}-x_{1}^{4})}+\frac{1}{5108x_{1}^{2}}-1\leq 0$,

                                $g_{3}(\vec{x})=1-\frac{140.45x_{1}}{x_{2}^{2}x_{3}}\leq 0$,

                                $g_{4}(\vec{x})=\frac{x_{1}+x_{2}}{1.5}-1\leq 0$,

Variable range 0.05≤*x*_1_≤2, 0.25≤*x*_2_≤1.30, 2≤*x*_3_≤15,

On the basis of IEO and EO, the tension/compression spring problem is optimized and the values of relevant parameters are obtained. The optimization results are compared with 10 algorithms in other literatures. The detailed information is shown in Table 12. And the optimal cost of IEO and comparison algorithms are shown in Fig 22.

**Table 12:** Comparison of result on tension/compression spring design problem.

Algorithm	Optimal values for variables			Optimal cost
	*d*	*D*	*N*	
IEO	**0.0516704**	**0.356269**	**11.3153**	**0.012665**
EO	0.0528	0.38404	9.8503	0.012687
BGRA [59]	0.0516747	0.3563726	1.309229	0.012665237
NM-PSO [60]	0.051620	0.355498	11.3333272	0.0126706
ES [43]	0.051643	0.355360	11.397926	0.012698
IHS [61]	0.0511543	0.3498711	12.0764321	0.0126706
AFA [44]	0.0516674837	0.3561976945	11.3195613646	0.0126653049
LSA-SM [45]	0.05170453	0.3570899	11.26718	0.01266524
EHO [52]	0.053666	0.406156	8.887284	0.012736
BWOA [62]	0.051602	0.357488	11. 24 41198	0.0126654
GSA [63]	0.050276	0.323680	13.525410	0.0127022
HS [61]	0.051609	0.354714	11.410831	0.0126702

As can be seen from Table 12, compared with other algorithms, the spring weight obtained by IEO algorithm is 0.012665. In general, IEO algorithm can effectively obtain the optimal solution of engineering problems and get the best parameter values.

### 4.4. Three-bar truss design problem

**Fig 23:**
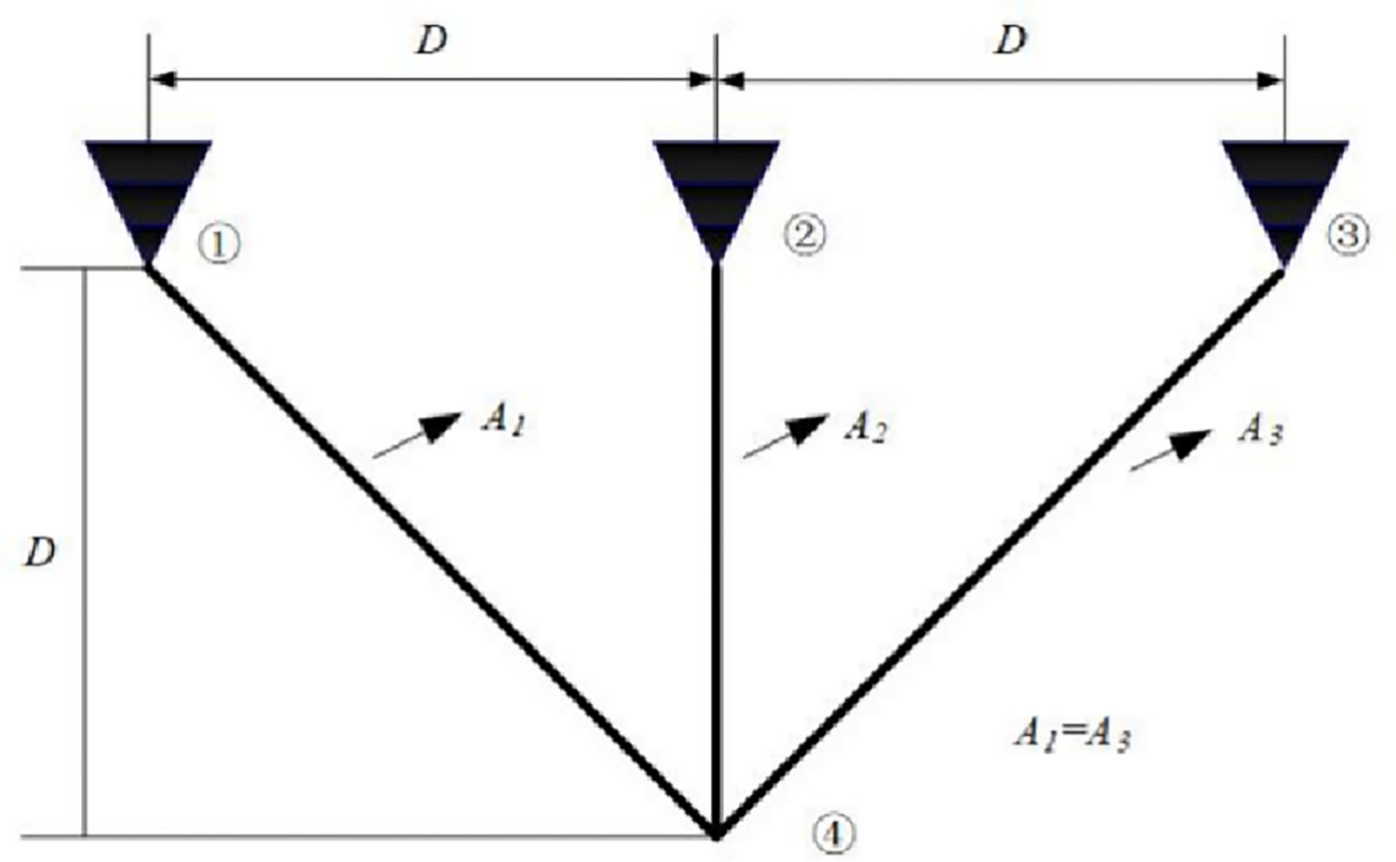
Three-bar truss design problem.

The problem of three-bar truss is a common application problem in civil engineering field [64], and its optimization purpose is to minimize the weight of the three-bar truss. This engineering problem includes two core parameters: *A*_1_ and *A*_2_ [65]. The details are shown in Fig 23.

The mathematical formula of this problem is expressed as follows:

Consider                $\vec{x}=[x_{1}\;x_{2}]=[A_{1}\;A_{2}]$,

Minimize                $f(\vec{x})=(2\sqrt{2}x_{1}+x_{2})*l$

Subject to                $g_{1}(\vec{x})=\frac{\sqrt{2}x_{1}+x_{2}}{\sqrt{2}x_{1}^{2}+2x_{1}x_{2}}P-\sigma \leq 0$,

                                $g_{2}(\vec{x})=\frac{x_{2}}{\sqrt{2}x_{1}^{2}+2x_{1}x_{2}}P-\sigma \leq 0$,

                                $g_{3}(\vec{x})=\frac{1}{\sqrt{2}x_{2}+x_{1}}P-\sigma \leq 0$,

Variable range 0≤*x*_1_, *x*_2_≤1

                                *l* = 100*cm*, *P* = 2 *km*/*cm*^2^, *σ* = 2 *km*/*cm*^2^.

**Fig 24:**
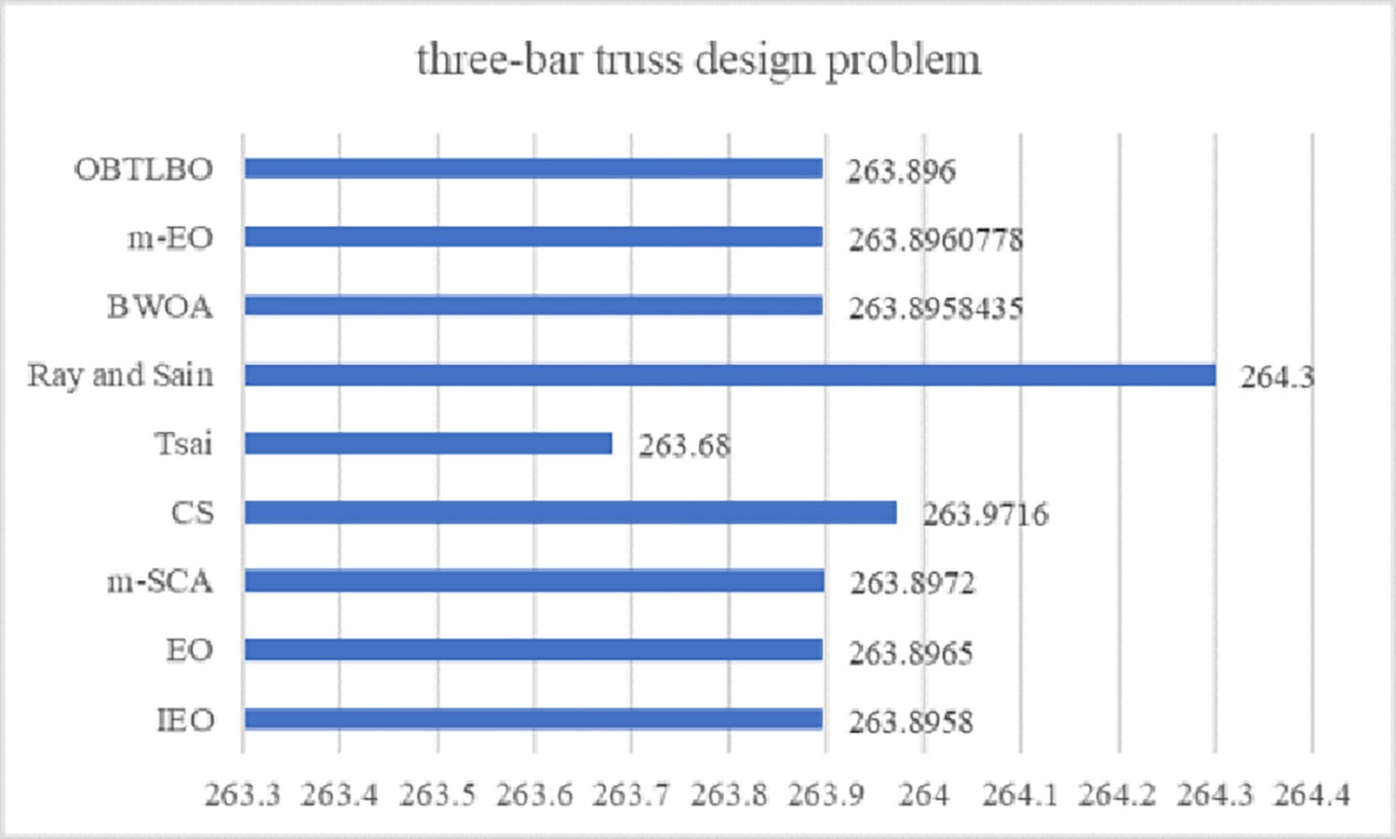
The optimal cost of three-bar truss design problem.

The IEO algorithm is applied to the three-bar truss problem, and the experimental results are compared with 8 other algorithms in other literatures. The results are shown in Table 13. Compared with other algorithms, it is obvious that IEO obtains the optimal solution for solving three-bar truss problem. It can also be seen from Fig 24 that IEO also has superior performance in solving practical engineering problems.

**Table 13:** Comparison of result on three-bar truss design problem.

Algorithm	Optimal values for variables		Optimal cost
	*A*_*1*_	*A*_*2*_	
IEO	**0.78868**	**0.40822**	**263.8958**
EO	0.7896	0.40563	263.8965
m-SCA [66]	0.81915	0.36956	263.8972
CS [67]	0.78867	0.40902	263.9716
Tsai [68]	0.788	0.408	263.68
Ray and Sain [64]	0.795	0.395	264.3
BWOA [62]	0.788666327	0.408273202	263.8958435
m-EO [1]	0.78834565	0.40918256	263.89607783
OBTLBO [19]	0.78909	0.40706	263.89600

### 4.5. Speed reducer design problem

**Fig 25:**
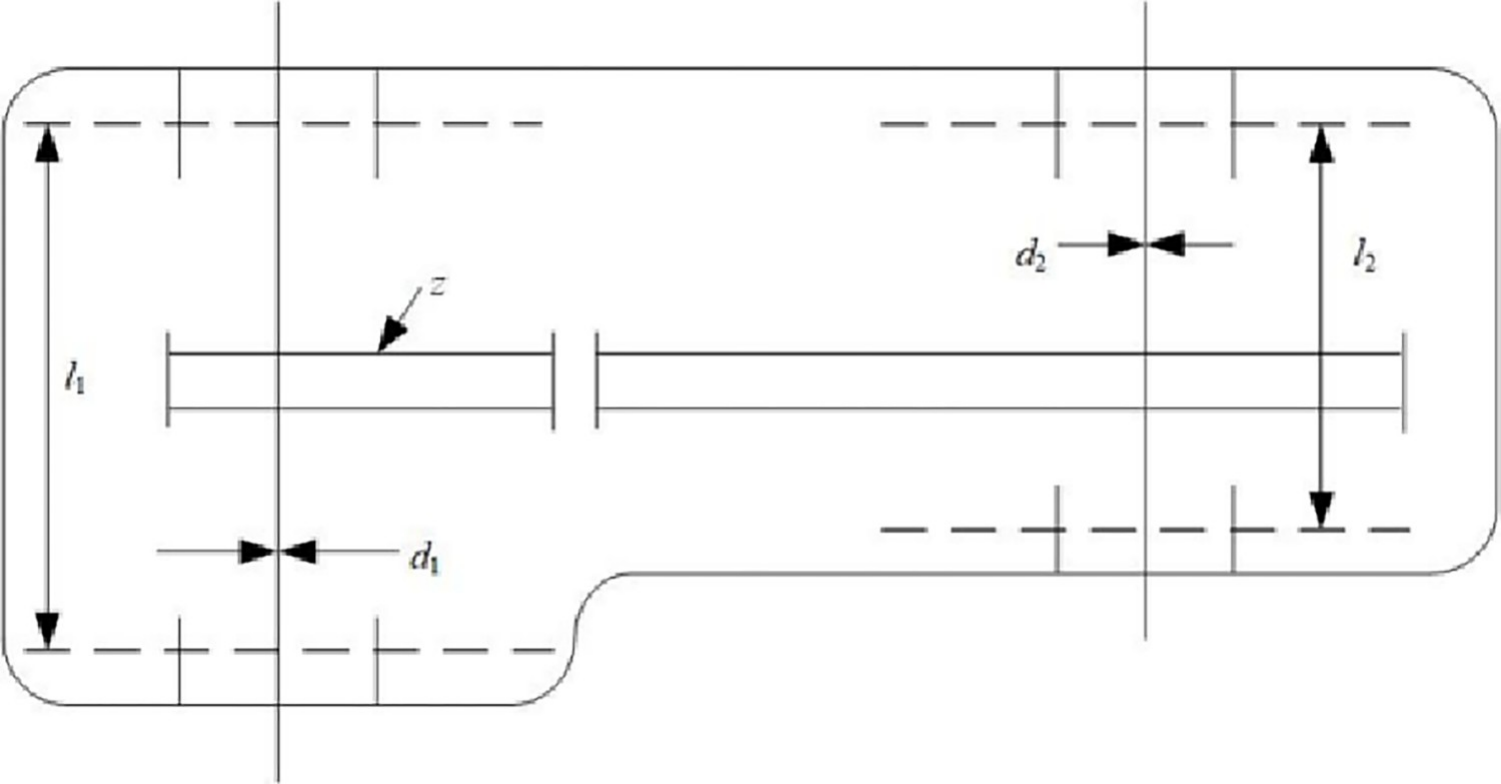
Speed reducer design problem.

Speed reducer problem is an engineering problem with complex constraints, and its optimization purpose is to minimize the weight of speed reducer itself. The constraint variables are shown in Fig 25.

The mathematical model of speed reducer is as follows:

Consider                $\vec{x}=[x_{1}\;x_{2}\;x_{3}\;x_{4}\;x_{5}\;x_{6}\;x_{7}]=[b\;m\;z\;l_{1}\;l_{2}\;d_{1}\;d_{2}]$

Minimize                $f(\vec{x})=0.7894x_{2}^{2}x_{1}(14.9334x_{3}-43.0934+3.3333x_{3}^{2})+0.7854(x_{5}x_{7}^{2}+x_{4}x_{6}^{2})-1.508x_{1}(x_{7}^{2}+x_{6}^{2})+7.477(x_{7}^{3}+x_{6}^{3})$

Subject to                $g_{1}(\vec{x})=-x_{1}x_{2}^{2}x_{3}+27\leq 0$

                                $g_{2}(\vec{x})=-x_{1}x_{2}^{2}x_{3}^{2}+397.5\leq 0$

                                $g_{3}(\vec{x})=-x_{2}x_{6}^{4}x_{3}x_{4}^{-3}+1.93\leq 0$

                                $g_{4}(\vec{x})=-x_{2}x_{7}^{4}x_{3}x_{5}^{-3}+1.93\leq 0$

                                $g_{5}(\vec{x})=10x_{6}^{-3}\sqrt{16.91\times 10^{6}+{\left(745x_{4}x_{2}^{-1}x_{3}^{-1}\right)}^{2}}-1100\leq 0$

                                $g_{6}(\vec{x})=10x_{7}^{-3}\sqrt{157.5\times 10^{6}+{\left(745x_{5}x_{2}^{-1}x_{3}^{-1}\right)}^{2}}-850\leq 0$

                                $g_{7}(\vec{x})=x_{2}x_{3}-40\leq 0$

                                $g_{8}(\vec{x})=-x_{1}x_{2}^{-1}+5\leq 0$

                                $g_{9}(\vec{x})=x_{1}x_{2}^{-1}-12\leq 0$

                                $g_{10}(\vec{x})=1.5x_{6}-x_{4}+1.9\leq 0$

                                $g_{11}(\vec{x})=1.1x_{7}-x_{5}+1.9\leq 0$

Variable range 2.6≤*x*_1_≤3.6, 0.7≤*x*_2_≤0.8, 17≤*x*_3_≤28, 7.3≤*x*_4_, *x*_5_≤8.3, 2.9≤*x*_6_≤3.9, 5≤*x*_7_≤5.5

**Fig 26:**
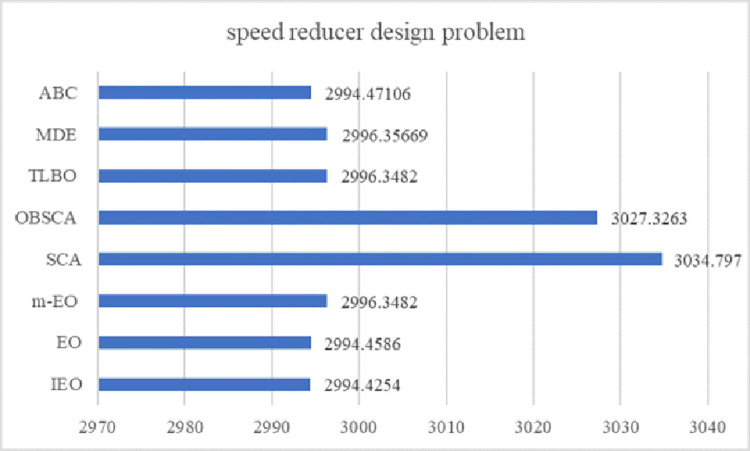
The optimal cost of speed reducer design problem.

On the basis of improved algorithm IEO, the speed reducer problem is optimized and the values of relevant parameters are obtained. The optimization results are compared with seven algorithms in other literatures. The details are shown in Table 14. To more clearly reflect the optimal cost of each algorithm, the Fig 18 is drawn. In addition, Fig 26 presents the optimal cost of IEO and comparison algorithms for speed reducer design problem.

**Table 14:** Comparison of result on speed reducer design problem.

Algorithm	Optimal values for variables							Optimal cost
	b	m	z	*l*_1_	*l*_2_	*d*_1_	*d*_2_	
IEO	**3.5000**	**0.7000**	**17.0000**	**7.30008**	**7.71532**	**3.35054**	**5.28665**	**2994.4254**
EO	3.5000	0.7000	17.0000	7.30366	7.71532	3.35055	5.28665	2994.4586
m-EO [19]	3.5000	0.7000	17.0000	7.3000	7.8000	3.3502	5.2867	2996.3482
SCA [19]	3.5198	0.7000	17.0000	7.3000	8.3000	3.4131	5.2919	3034.7970
OBSCA [19]	3.1507	0.7716	19.9472	7.7174	8.2332	3.5060	5.2938	3027.3263
TLBO [19]	3.5000	0.7000	17.0000	7.3000	7.8000	3.3502	5.2867	2996.3482
MDE [40]	3.50001	0.7000	17.0000	7.300156	7.800027	3.350221	5.286685	2996.35669
ABC [69]	3.5000	0.7000	17.0000	7.3000	7.715319	3.350214	5.286654	2994.47106

Compared with other algorithms, the improved algorithm IEO in this paper has higher accuracy in dealing with speed reducer engineering problem. In other words, The IEO algorithm find the best values for seven design variables to minimize the weight of speed reducer.

### 4.6. Optimal design of industrial refrigeration system

At present, energy saving and emission reduction work has become the focus of various fields. Industrial refrigeration system accounts for a large proportion of energy consumption, so it is necessary to optimize and control the industrial refrigeration system. Optimal design of industrial refrigeration system is an extremely complex engineering design problem, which has fourteen design variables and fifteen constraints. Its mathematical model is shown as follows:

Consider                $\vec{x}=[x_{1}\;x_{2}\;x_{3}\;x_{4}\;x_{5}\;x_{6}\;x_{7}\;x_{8}\;x_{9}\;x_{10}\;x_{11}\;x_{12}\;x_{13}\;x_{14}]$

Minimize                $f(\vec{x})=63098.88x_{2}x_{4}x_{12}+5441.5x_{2}^{2}x_{12}+115055.5x_{2}^{1.664}x_{6}+6172.27x_{2}^{2}x_{6}+63098.88x_{1}x_{3}x_{11}+5441.5x_{1}^{2}x_{11}+115055.5x_{1}^{1.664}x_{5}+6172.27x_{1}^{2}x_{5}+140.53x_{1}x_{11}+281.29x_{3}x_{11}+70.26x_{1}^{2}+281.29x_{1}x_{3}+281.29x_{3}^{2}+14437x_{8}^{1.8812}x_{12}^{0.3424}x_{10}x_{14}^{-1}x_{7}x_{9}^{-1}+20470x_{7}^{2.893}x_{11}^{0.316}x_{1}^{2}$

Subject to                $g_{1}(\vec{x})=1.524x_{7}^{-1}\leq 1$

                                $g_{2}(\vec{x})=1.524x_{8}^{-1}\leq 1$

                                $g_{3}(\vec{x})=0.07789x_{1}-2x_{7}^{-3}x_{9}-1\leq 0$

                                $g_{4}(\vec{x})=7.05305x_{9}^{-1}x_{1}^{2}x_{10}x_{8}^{-1}x_{2}^{-1}x_{14}^{-1}-1\leq 0$

                                $g_{5}(\vec{x})=0.0833x_{13}^{-1}x_{14}-1\leq 0$

                                $g_{6}(\vec{x})=47.136x_{2}^{0.333}x_{10}^{-1}x_{12}-1.333x_{8}x_{13}^{2.1195}+62.08x_{13}^{2.1195}x_{12}^{-1}x_{8}^{0.2}x_{10}^{-1}-1\leq 0$

                                $g_{7}(\vec{x})=0.04771x_{10}x_{8}^{1.8812}x_{12}^{0.3424}-1\leq 0$

                                $g_{8}(\vec{x})=0.0488x_{9}x_{7}^{1.893}x_{11}^{0.316}-1\leq 0$

                                $g_{9}(\vec{x})=0.0099x_{1}x_{3}^{-1}-1\leq 0$

                                $g_{10}(\vec{x})=0.0193x_{2}x_{4}^{-1}-1\leq 0$

                                $g_{11}(\vec{x})=0.0298x_{1}x_{5}^{-1}-1\leq 0$

                                $g_{12}(\vec{x})=0.056x_{2}x_{6}^{-1}-1\leq 0$

                                $g_{13}(\vec{x})=2x_{9}^{-1}-1\leq 0$

                                $g_{14}(\vec{x})=2x_{10}^{-1}-1\leq 0$

                                $g_{15}(\vec{x})=x_{12}x_{11}^{-1}-1\leq 0$

Variable range 0.001≤*x*_*i*_≤5, *i* = 1,…,14

**Fig 27:**
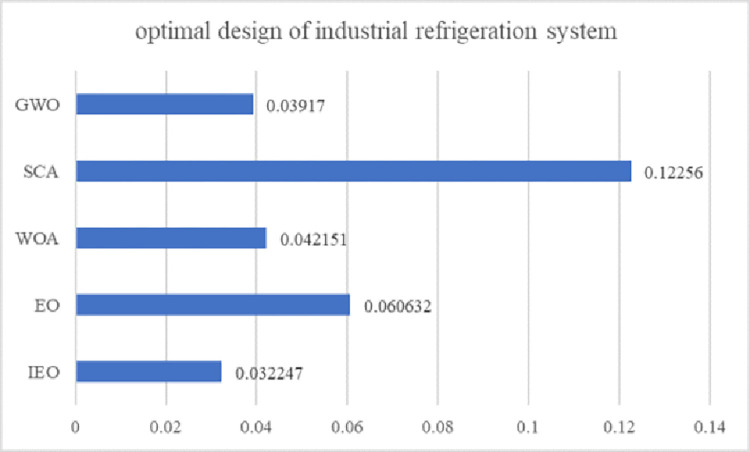
The optimal cost of optimal design of industrial refrigeration system.

Fourteen key variables of optimal design of industrial refrigeration system are optimized by IEO algorithm and the optimal values are obtained, and the results are compared with other meta-heuristic algorithms. Table 15 shows the lowest cost of each algorithm and the values of related variables. Fig 27 shows the optimal cost of the five algorithms for optimal design of industrial refrigeration system.

**Table 15:** Comparison of result on optimal design of industrial refrigeration system.

variable	algorithm				
	IEO	EO	WOA	SCA	GWO
*x*_1_	**0.001**	0.001	0.0010002	0.001	0.001
*x*_2_	**0.0010005**	0.0010003	0.0010079	0.0028508	0.0010782
*x*_3_	**0.0010001**	0.0010005	0.0010205	0.0044385	0.0010242
*x*_4_	**0.0010121**	0.0012186	0.0058931	0.0016035	0.0012434
*x*_5_	**0.0010002**	0.0011102	0.001	0.0015727	0.0019951
*x*_6_	**0.0010009**	0.0013208	0.0010007	0.001235	0.0010718
*x*_7_	**1.524**	1.5245	1.5246	1.7023	1.5243
*x*_8_	**1.524**	1.524	1.524	1.7463	1.5244
*x*_9_	**5**	5	4.9963	4.6685	4.9977
*x*_10_	**2**	3.0708	2.0001	2.3346	2.0099
*x*_11_	**0.0010001**	0.029739	0.0010077	0.0037313	0.0067319
*x*_12_	**0.001**	0.029569	0.0010007	0.002211	0.0066506
*x*_13_	**0.0072837**	0.043223	0.0050226	0.0046903	0.017752
*x*_14_	**0.087436**	0.51759	0.060171	0.052574	0.21302
optimal value	**0.032247**	0.060632	0.042151	0.12256	0.03917

As can be seen from the optimization results in Table 15, IEO algorithm still has good performance in dealing with highly complex engineering design problems. Compared with the original EO algorithm, the optimal value obtained by IEO algorithm is improved by several orders of magnitude. In general, for engineering problems with fourteen objective variables and constraints, IEO algorithm can also get the optimal value.

In this section, six engineering optimization problems of pressure vessel, welded beam, tension/compression spring, three-bar truss, speed reducer and optimal design of industrial refrigeration system with constraint state are solved by IEO algorithm, and the design scheme given by IEO is compared with the scheme proposed by the algorithms in the existing literature. The comparison results show that the design cost of IEO is much lower than the original EO algorithm and other comparison algorithms, and it is an algorithm that can effectively solve engineering optimization problems. At the same time, the good optimization results also show that IEO has better optimization efficiency and performance in practical application. Each engineering optimization problem has different variables and constraints. According to the number of variables and constraints, it can be divided into engineering problems of different complexity. The mathematical model of each engineering optimization problem corresponds to the actual engineering problems in real life. By using IEO to solve engineering optimization problems, it shows that the algorithm has superior robustness, which indicates that IEO can be applied to practical engineering problems in the future.

Through the above numerical experiments and the application of six engineering problems, we can see that the IEO algorithm has excellent performance. Specifically, the effectiveness of IEO is measured by three criteria: mean value, standard deviation and running time. For F14-F23 in the 23 benchmark function sets, these functions belong to 2-dimensional, 4-dimensional and 6-dimensional functions, and the optimization effect of IEO is not outstanding. However, for functions F1-F13 with a dimension of 30, the solution efficiency of IEO is obviously improved. And for the complex function sets CEC2017 and CEC2019 in later sections, the convergence accuracy of IEO is improved. In addition, through Friedman test and Wilcoxon rank sum test, the significance of IEO algorithm can be clearly seen from the perspective of statistics. In section 4, IEO is used to solve six engineering problems of different complexity. By comparing the optimal cost with other algorithms, the practicability of IEO in engineering problems is known, which further verifies the effectiveness of IEO algorithm. Overall, the superiority of IEO is analyzed from different perspectives through complex numerical experiments and the application of algorithms to engineering problems.

### 5.Conclusion

In this paper, a multi-strategy improved Equilibrium Optimizer (IEO) is proposed to solve numerical optimization and engineering problems. Tent mapping is used to initialize the population and produce the initial solution with rich diversity, which lays a good foundation for the global search of the search population in space. A nonlinear time parameter strategy is also introduced into the update equation of the algorithm, which dynamically coordinates the exploration and exploitation phase of IEO algorithm. The Lens Opposition‑based Learning (LOBL) strategy is adopted in the late iteration of the algorithm to improve the diversity of the population and prevent the algorithm form falling into local optimal. Simulation experiments are carried out by using 23 classical functions, IEEE CEC2017 and IEEE CEC2019. The experimental results show that compared with the other six meta-heuristic algorithms, the improved IEO algorithm has obvious advantages in solving accuracy and convergence speed. In addition, the stability and effectiveness of IEO are proved from different perspectives by Friedman statistical test and Wilcoxon rank sum test. Finally, IEO is applied to six engineering design problems: the pressure vessel problem, the welded beam problem, the tension /compression spring problem, the three-bar truss problem, the speed reducer problem and optimal design of industrial refrigeration system. The research results show that the improved IEO algorithm has good optimization efficiency when solving practical application problems. In the future, the IEO will be tried to combine with other meta-heuristic algorithms to better improve the performance. The IEO may be implemented on complex real-world application problems, such as feature selection and robot path planning. The IEO also can be applied to multi-objective problems and more complex practical engineering problems.
